# Study of Direct *N*^7^ Regioselective *tert*-Alkylation of 6-Substituted Purines and Their Modification at Position *C*^6^ through O, S, N, and C Substituents

**DOI:** 10.1021/acsomega.4c00068

**Published:** 2024-04-06

**Authors:** Filip Nevrlka, Adam Bědroň, Michal Valenta, Lenka Tranová, Jakub Stýskala

**Affiliations:** Department of Organic Chemistry, Faculty of Science, Palacký University, 17. Listopadu 12, 771 46 Olomouc, Czech Republic

## Abstract

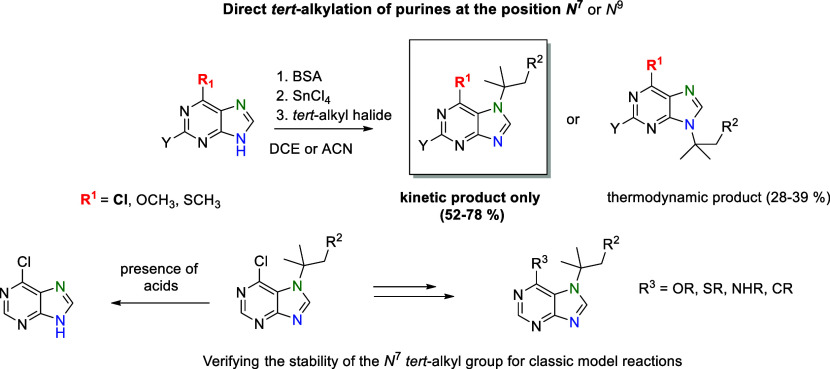

A new *N*^7^ direct regioselective method allowing the introduction of *tert*-alkyl groups into appropriate 6-substituted purine derivatives is developed. This method is based on a reaction of *N*-trimethylsilylated purines with a *tert*-alkyl halide using SnCl_4_ as a catalyst. In this work, we study the structure and optimal reaction conditions leading to the *N*^7^ isomer and in some cases also to the *N*^9^ isomer. The main goal is devoted to preparing 7-(*tert*-butyl)-6-chloropurine as a suitable compound for other purine transformations. The stability of the *tert*-butyl group at the *N*^7^ position is tested for classic model reactions, leading to the preparation of new 6,7-disubstituted purine derivatives, which is also interesting from the point of view of possible biological activity.

## Introduction

Purine is a heterocyclic compound that is most commonly found in nature and is often found in several molecules with proven biological properties.^1,2^ For several decades, attention has therefore been focused on the chemistry of purines to obtain biologically active compounds. New chemical reactions allowing its modification can still be found. These reactions may involve, for example, regioselective substitution at the *N*^7^ position of the purine ring, which represents a poorly explored area of purine chemistry and related biological activity.

Although *N*^7^-substituted purine derivatives are less widespread than their *N*^9^ analogues, several biologically interesting compounds can be found among them. For example, pseudovitamin B12 or raphanatin, which has cytokinin activity,^3^ can be considered to be an *N*^7^ nucleoside in nature. Interesting cytotoxic activity^4,5^ or inhibitory activity of butyrylcholinesterase has also been reported for *N*^7^ nucleosides.^6−8^ Compounds with antiviral activity can also be found among *N*^7^-alkylated purines.^9−14^ More specifically, some *N*^7^-substituted adenines showed antiviral^15,16^ and anticancer activities.^17,18^ Some *N*^7^-substituted 6-mercaptopurines showed cytostatic activity,^19,20^ and 7-methylxanthine was discovered to be a DNA gyrase inhibitor.^21^

Regarding the preparation of *N*^7^-alkylated purines, there are several ways to introduce an alkyl group at the *N*^7^ position of the purine ring. Unfortunately, the methods are complicated, or a mixture of corresponding isomers is formed, where the thermodynamically more stable *N*^9^ regioisomer usually predominates, while the *N*^7^ isomer occurs as a side product.

**Scheme 1:**
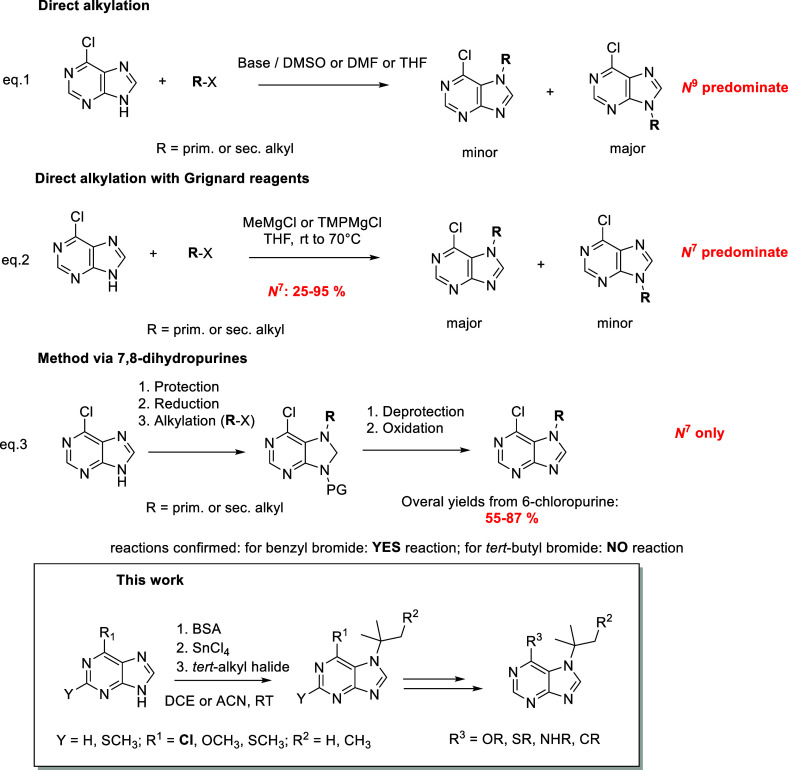
Preparation of *N*^7^-Alkylated 6-Chloropurines

One method is based on alkylation of purine derivatives with alkyl halides under basic conditions (e.g., 6-chloropurine^22−27^). This direct alkylation usually leads to a mixture of *N*^7^/*N*^9^ derivatives and is suitable when both isomers are of interest (Scheme 1, eq 1).

A second method using Grignard reagents is similar to the previous method, but *N*^7^ isomers are usually favored (Scheme 1, eq 2).^28^

Very unambiguous is the method conducted via 7,8-dihydropurines. This method is based on protection of the *N*^9^ position and reduction and regioselective alkylation at position *N*^7^, followed by deprotection and reoxidation (Scheme 1, eq 3).^29,30^

Additional methods are based on the cyclization reaction of appropriate imidazole^31,32^ or pyrimidine derivatives.^33−35^ These methods represent unambiguous regioselectivity but are of multistep and very laborious.

Other methodologies for regioselective *N*^7^ alkylation of purines are limited to allylation of 6-halopurines in the presence of a cobalt complex^36^ or alkylation of some *N*^9^-substituted purines, followed by selective cleavage of labile groups from formed purinium salts.^10,37−40^

In general, 6-chloropurine (**1**) is a very useful intermediate for further derivatization and biological research. We decided to expand the scope of the less common *N*^7^ purine regioselective substitution with *tert*-alkyl groups, which could not be introduced by the methods described in the literature. The main goal is to develop a new method enabling the easy introduction of a *tert*-alkyl group into 6-chloropurine at the *N*^7^ position and to verify its stability under different reaction conditions, as well as to prepare new 6,7-purine disubstituted derivatives containing different substituents connected via O, S, N, and C atoms that may be of interest in terms of possible biological activity (Scheme 1, this work).

## Results and Discussion

The reported alkylation methods (Scheme 1, eqs 1–3) employ only primary or secondary alkyl halides, not tertiary. First, we successfully experimentally verified these methods from the literature for the described substances and then applied them to the *tert*-butyl group but without success. No desired reaction occurred.

For the introduction of the *tert*-butyl group, we were inspired by the Vorbrüggen (silylation) method,^41^ which is generally used for the preparation of purine and pyrimidine nucleosides. This method allows the preparation of mainly thermodynamically favored *N*^9^ isomers in purine chemistry. However, there are also reactions where the predicted regioselectivity decreases^8,42,43^ or is reversed in favor of *N*^7^ isomers.^44−46^

Based on previous experience with the Vorbrüggen method,^46^ we successfully used a *tert*-alkyl halide instead of the protected sugar for the reaction with the purine derivatives. Initial pilot studies were performed with 6-chloropurine (**1**), the most interesting derivative, and *tert*-butyl bromide (*tert*-BuBr) to obtain 7-(*tert*-butyl)-6-chloropurine (**2**) with maximum possible regioselectivity and yield.

We made several assumptions regarding how to obtain a kinetic *N*^7^ product. These included assumptions on the influence of temperature, time, solvent [1,2-dichloroethane (DCE), acetonitrile (ACN)], type of Lewis acid, and other alkyl halides (Table 1).

**Table 1:**
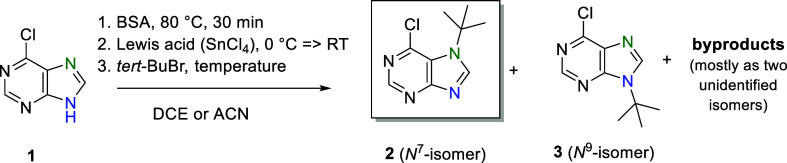
Optimization of the *tert*-Butylation Reaction of 6-Chloropurine (**1**)

entry	solvent	SnCl_4_ (equiv)	*tert*-BuBr (equiv)	reaction time (h)	temperature	1:2:3:byproducts %^a^
1	DCE	0	3	19	RT	100:0:0:0
2	DCE	1	1	3	RT	90:10:0:0
3	DCE	1	1	19	RT	83:17:0:0
4	DCE	1.2	1.5	3	RT	83:17:0:0
5	DCE	1.2	1.5	19	RT	60:40:0:0
6	DCE	1.2	3	3	RT	70:30:0:0
7	DCE	1.2	3	19	RT	40:60:0:0
8	DCE	2.1	1.5	3	RT	55:45:0:0
9	DCE	2.1	1.5	19	RT	50:50:0:0^b^
10	DCE	2.1	3	3	RT	25:75:0:0
**11**	**DCE**	**2.1**	**3**	**19**	**RT**	**13:87:0:0**^**c**^
12	DCE	2.1	3	48	RT	12:86:0:2
13	DCE	2.1^d^	1.5	19	RT	55:45:0:0
14	DCE	2.1^d^	3	19	RT	34:66:0:0
15^g^	DCE	2.1	3	19	RT	100:0:0:0
16	ACN	2.1	1.5	3	RT	38:62:0:0
17	ACN	2.1	1.5	19	RT	37:63:0:0
18	**ACN**	**2.1**	**3**	**3**	**RT**	**11:87:0:2**^h^
19	ACN	2.1	3	19	RT	10:84:0:6
20	ACN	2.1	3	48	RT	15:73:2:10
21	DCE	2.1	3	3	50 °C	12:87:1:0
22	DCE	2.1	3	19	50 °C	13:79:3:5
23	ACN	2.1	3	3	50 °C	32:48:5:15
24	ACN	2.1	3	19	50 °C	15:5:30:50
25	**ACN**	**2.1**	**3**	**5**	**80°C**	**15:0:55:30**^i^

a Based on LC/MS analysis.

b The isolated yield of **2** was 40%.

c The isolated yield of **2** was 75%.

d TiCl_4_ was used as a catalyst.

f The isolated yield of **2** was 43%.

g The reaction was carried out without prior silylation.

h The isolated yield of **2** was 78%.

i The isolated yield of *N*^9^-isomer **3** was 39%. For entries 18 and 25, see the Supporting Information.

In general, since the *N*^7^-substituted compounds are described as kinetically favorable products, the reactions were optimized by performing them under kinetically controlled conditions, i.e., mainly at room temperature, with regard to the optimal reaction time to capture the kinetic product and achieve the maximum possible conversion of the starting compounds without the formation of side products.

The Lewis acids most widely used as Vorbrüggen reaction catalysts include trimethylsilyl trifluoromethanesulfonate (TMSOTf), TiCl_4_, and SnCl_4_. The best results of the *tert*-butylation reaction of 6-chloropurine were obtained with the use of SnCl_4_ in the amount of 2.1–1 equiv of purine derivative. Similar amounts of catalyst appear in the literature for the Vorbrüggen reaction, which requires the formation of a cation from a saccharide and complexation with the purine derivative. A lower amount of catalyst had a considerable effect on the conversion of the starting material (Table 1, entries 2–7). Without a catalyst, the reaction did not occur at all (Table 1, entry 1). When TiCl_4_ was used, the reaction also occurred, but the conversion was lower (Table 1, entries 13–14). In contrast, when using TMSOTf or other tested Lewis acids [FeCl_3_, ZnCl_2_, AlCl_3_, BF_3_, Et_2_O, and Ti(*i*PrO)_4_], the reactions did not occur at room temperature. Additionally, no reaction was observed if the previous silylation with *N*,*O*-bis(trimethylsilyl)acetamide (BSA) was omitted (Table 1, entry 15).

The degree of conversion was also affected by the amount of *tert*-butyl bromide used. For successful reactions, 3 equiv of *tert*-butyl bromide was a sufficient quantity. Although the degree of conversion was not quite complete, the unreacted starting compound **1** could be easily removed by an extraction process after the reaction in the water–NaHCO_3_ system to obtain the desired crude product of high purity.

The solvent used had a significant effect on the rate of the kinetic *tert*-butylation reaction. DCE and more polar ACN, both widely used in the Vorbrüggen silylation method, were tested. The equilibrium was established faster with ACN (3 h) than with DCE (19 h) (compare Table 1, entries 10 and 11 with entries 18 and 19). In both cases, a longer reaction time (48 h) caused the gradual formation of additional isomers [detected by liquid chromatography–mass spectrometry (LC/MS)], which was significantly faster when using ACN (compare Table 1, entry 12 with entry 20).

**Scheme 2:**
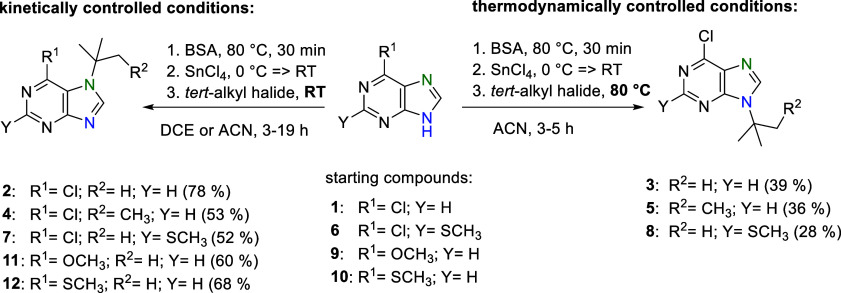
Preparation of *N*^7^/*N*^9^-(*tert*-Alkylated) Purine Derivatives

The effect of temperature was also significant but expected in the described reactions. The reaction carried out at 50 °C for both solvents caused rapid formation of *N*^7^-isomer **2**, but along with other isomers. Again, in the case of ACN, the formation of other isomers was faster, and after 19 h of heating, the portion of *N*^7^-isomer **2** started to change in favor of starting compound **1** and other isomers, where the thermodynamically more stable *N*^9^-isomer **3** predominated (compare Table 1, entry 22 with entry 24). Based on these results, we performed the silylation *tert*-butylation reaction with **1** in ACN at 80 °C to obtain *N*^9^-isomer **3**. After 5 h of heating, no *N*^7^-isomer **2** was present, and the reaction mixture contained predominant *N*^9^-isomer **3** in addition to other byproducts (Table 1, entry 25). In a similar way, other *N*^9^-(*tert*-alkylated) 6-chloropurine derivatives **5** and **8** were obtained in yields of 28–39% (Scheme 2). This method can also represent the possibility of preparing *tert*-alkylated *N*^9^ isomers that cannot be obtained by classical direct alkylation (Scheme 1, eq 1).

In addition to the reaction with 6-chloropurine (**1**), we tested other purine derivatives to verify whether the substituent on the purine ring plays a role in the *tert*-butylation reaction. No reaction was observed when unsubstituted purine, 6-methylpurine, 2-chloropurine, and 6-(dimethylamino)purine were used. In contrast, 6-methoxy (**9**), 6-methylthiopurine (**10**), and 6-chloro-2-methylthiopurine (**6**) readily underwent *N*^7^ regioselective *tert*-butylation to obtain *N*^7^-isomers **7**, **11**, and **12** (Scheme 2). The substituents at the *C*^6^ position clearly play an important role during the regioselective reaction.

We were curious if the method developed for the *N*^7^ regioselective *tert*-butylation of 6-chloropurine (**1**) would be useable for other types of alkyl halides. Primary (benzyl bromide, allyl bromide, ethyl bromide, methyl iodide), secondary (isopropyl bromide), and tertiary (neopentyl bromide) halides were tested. Unfortunately, this silylation method failed for primary and secondary alkyl halides. A very low conversion, of approximately 10%, was observed for benzyl bromide in obtaining the corresponding *N*^7^ isomer, which was identified by LC/MS analysis of the reaction mixture using appropriate *N*^7^-benzylated^28^ and *N*^9^-benzylated^47^ standards prepared by different described methods. This example suggests that other stabilized carbocations may influence purine substitution. However, by using *tert*-neopentyl bromide, the presented method was confirmed to be useable for other *tert*-alkyl halides. Under kinetically controlled conditions, the *N*^7^-isomer **4** was obtained (Scheme 2).

**Figure 1:**
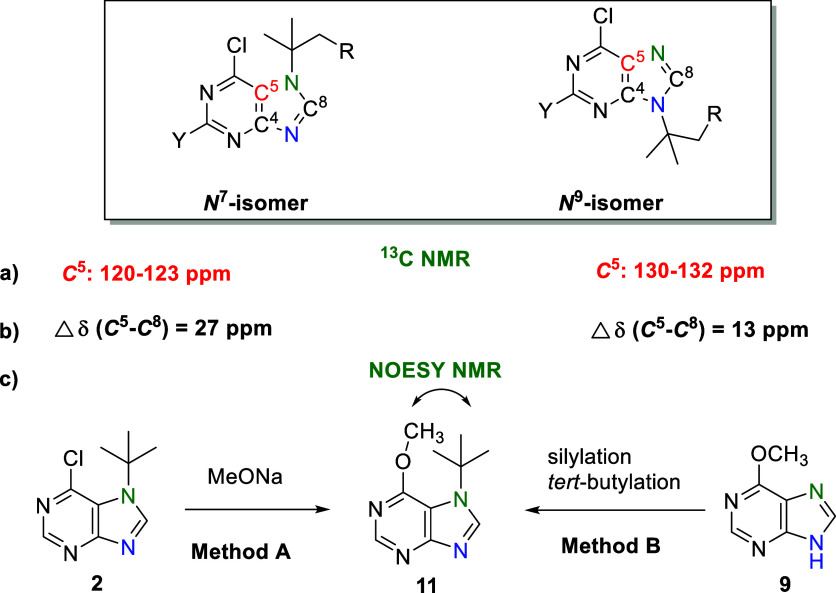
Resolution of *N*^7^/*N*^9^ regioisomers based on (a) chemical shift of the *C*^5^ carbon atom, determined using HMBC and HSQC experiments; (b) difference between the chemical shifts of the *C*^5^ and *C*^8^ carbon atoms; (c) NOESY NMR experiment.

7-(*tert*-Butyl)-6-chloro-7*H*-purine (**2**) is a new compound. The position of the *tert*-butyl group was confirmed in several ways using ^13^C NMR spectroscopy, where carbon structural assignments of **2** and **3** were made based on heteronuclear multiple-bond correlation (HMBC) and heteronuclear single-quantum coherence (HSQC) experiments. To differentiate between the two isomers, the key attribute is the chemical shift of the *C*^5^ carbon atom of the 6-chloropurine ring. Based on published values, *N*^9^-alkylated or *N*^9^-glycosylated 6-chloropurine derivatives show a chemical shift for the *C*^5^ atom of approximately 132 ppm.^27,48^ In contrast, the *C*^5^ chemical shift of the *N*^7^ isomers of 6-chloropurine is more shielded and shows a lower value of approximately 123 ppm.^28−30,35^ This is in accordance with our observations for all 6-chloropurine isomers **2, 4**, and **7** and **3, 5**, and **8** (Figure 1).

Generally, a reliable tool for the resolution of *N*^7^ and *N*^9^ purine isomers is a method based on the relative difference between the chemical shifts of *C*^5^ and *C*^8^ carbon atoms. For *N*^7^ acylpurines, the difference is large, while for *N*^9^ isomers, the difference Δδ is much smaller.^49,50^ This is also known to hold for *N*^7^/*N*^9^ 6-chloropurine nucleosides.^46^ The observed difference results are similar for our prepared *N*^7^-isomer **2** (Δδ = 27) and *N*^9^-isomer **3** (Δδ = 13).

The *N*^7^-regioisomer **2** was also unambiguously determined by an NMR nuclear Overhauser effect spectroscopy (NOESY) experiment after derivatization at position *C*^6^ by a methoxy group to obtain compound **11** (Figure 1, Method A), where the interaction of *tert*-butyl hydrogen atoms and the methoxy group was observed. Similar cross peaks are apparent in the NOESY spectra for 6-methoxy **11** (Figure 1, Method B) and 6-methylthio derivative **12** prepared by silylation methods from the corresponding precursors **9** and **10**.

In addition, the *N*^9^ position of the *tert*-butyl group of *N*^9^-isomer **3**, which was also previously prepared by a different method via ring closure of the imidazole ring,^51^ was confirmed by comparing the published ^1^H NMR and melting point data, which were consistent with the data of our product **3** prepared by silylation *tert*-butylation under thermodynamically controlled conditions.

**Scheme 3:**
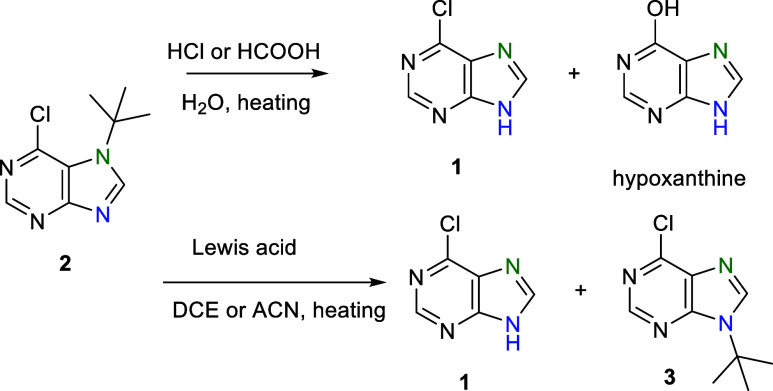
Instability of the *N*^7^-(*tert*-Butyl) Group of **2** in the Presence of Acidic Compounds

Considering the possible lability of the *tert*-alkyl group, we decided to perform the following tests. We found that the *tert*-butyl group at position *N*^7^ of compound **2** is stable in basic conditions, but in the presence of aqueous mineral acids (HCl, HCOOH) or Lewis acids, it is unstable, contrary to *N*^9^-isomer **3**, which is stable in all cases. The stability of *N*^7^-isomer **2** in the presence of Lewis acid was tested with SnCl_4_ (1 equiv) in DCE and ACN at 50 °C. Heating in DCE caused 20% conversion to 6-chloropurine (**1**) after 48 h. Only traces of the *N*^9^-isomer **3** were detected. When using ACN as a solvent, a mixture of starting compound **1**, *N*^7^-isomer **2**, and *N*^9^-isomer **3** as major compounds was detected after 21 h. Additionally, other minor, probably *N*^1^ and *N*^3^, *tert*-butyl isomers of 6-chloropurine were detected by LC/MS analysis. Complete conversion of the starting *N*^7^-isomer **2** was achieved after 48 h, where 6-chloropurine (**1**) and the thermodynamically most stable *N*^9^-isomer **3** were observed as the predominant products. Similar results were observed using TMSOTf (2 equiv) after heating at 80 °C for 1 h in ACN, where a mixture of compounds **1** and **3** was observed at a ratio of 1:3 (Scheme 3).

**Scheme 4:**
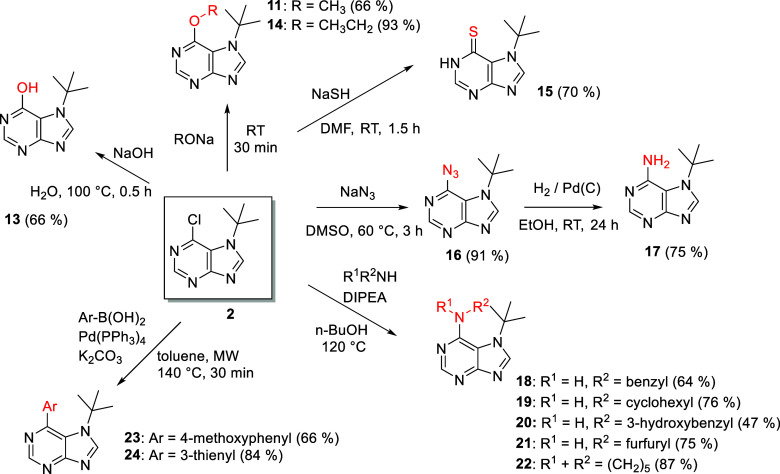
Modification of 7-(*tert*-Butyl)-6-chloro-7*H*-purine (**2**) at Position *C*^6^

Prepared 7-(*tert*-butyl)-6-chloropurine (**2**) was further used for the *C*^6^ modification (Scheme 4). First, we focused on the preparation of hypoxanthine analogue **13**, which was prepared by the alkaline hydrolysis of **2**. Because of the low stability of *tert*-butyl at position *N*^7^ in acidic solutions, we cannot afford to carry out this hydrolysis with HCOOH, which has been utilized for analogous 7-alkyl-6-chloropurines, where alkyl represents methyl, allyl, propargyl, ester, and keto groups.^52^ From this point of view, the *tert*-butyl group is specific. Analogically, using the corresponding alkoxides, methoxy **11** and ethoxy **14** derivatives were prepared under mild conditions and in high yields. These were used for NMR NOESY experiments to determine the position of the *tert*-butyl group. The sulfur analogue of hypoxanthine **15** was prepared by treating starting compound **2** with a solution of NaSH in dimethylformamide (DMF) at room temperature, indicating complete conversion after 90 min. The first chromatographic separation of **15** failed due to partial oxidation to disulfide, so rapid extraction of the product under inert conditions to obtain the solid product was preferable.

**Scheme 5:**
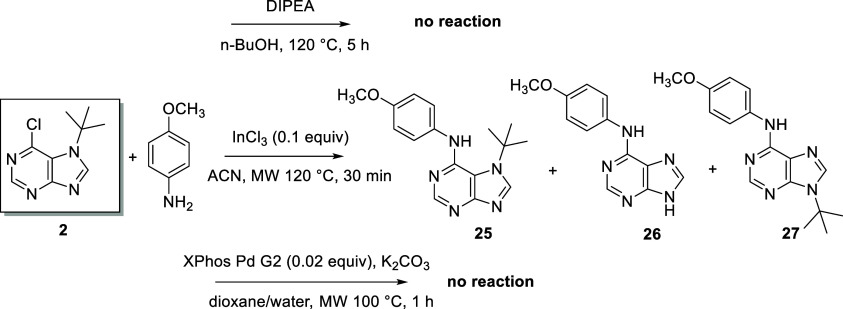
Illustration of the Problem of Substituting a Chlorine Atom with a Less Nucleophilic Amine

Adenine derivative **17** was prepared by a two-step protocol from azide **16**, which was smoothly prepared with sodium azide in dimethyl sulfoxide (DMSO) at 60 °C. Reduction of azide **16** was carried out by palladium-catalyzed hydrogenation, which offers a simple and efficient method under slightly elevated hydrogen pressure without the undesired influence on the *tert*-butyl group. Other *N*-substituted adenine derivatives (**18**–**22**) were prepared by classical nucleophilic substitution of the *C*^6^ chlorine atom of **2** with structurally different amines in the presence of *N*,*N*-diisopropylethylamine (DIPEA) in *n*-BuOH at 120 °C (Scheme 4). A similar reaction with a less nucleophilic amine (4-methoxyaniline) failed under the conditions described above, and no conversion was observed in the Buchwald–Hartwig amination. A mixture of starting compound **2**, desired compound **25**, de-(*tert*-butylated) derivative **26**, and less polar *N*^9^ derivative **27** was detected by LC/MS analyses according to the described coupling protocol using InCl_3_ as a catalyst.^53^ This reaction confirmed the instability of the *tert*-butyl group at the *N*^7^ position using acidic compounds (Scheme 5). However, the *tert*-butyl group was retained under Suzuki cross-coupling conditions for two model compounds **23** and **24** during C–C bond formation from the corresponding boronic acids when Pd(PPh_3_)_4_ was used as a catalyst under microwave (MW) irradiation (Scheme 4).

## Conclusions

By studying the reaction conditions (catalyst type, solvent, time, and temperature), we developed a new method enabling direct regioselective *N*^7^-(*tert*-alkylation) of *C*^6^-substituted purines under kinetically controlled conditions. This reaction is highly regioselective for the *tert*-alkyl group, where there is a specific substituent at position *C*^6^. The method mentioned above enables the preparation of even *N*^9^ isomers under thermodynamic conditions. Apart from the presence of acidic compounds causing cleavage of the *N*^7^-(*tert*-butyl) group, this group is stable, and using chlorine precursor **2**, various derivatives substituted at position *C*^6^ through O, S, N, and C atoms can be obtained as new 6,7-disubstituted purines.

## Experimental Section

### General Methods

All reagents were purchased from commercial suppliers and used without purification. Solvents were dried according to standard procedures and stored with molecular sieves 3A. Reactions were monitored by LC/MS analyses using a UPLC Waters Acquity system equipped with PDA and QDa detectors. The system contained an XSelect HSS T3 (Waters) 3 × 50 mm C18 reverse-phase column XP (2.5 μm particles). Mobile phases: 10 mM ammonium acetate in HPLC grade water (A) and gradient grade ACN for HPLC (B). A gradient was mainly formed from 20 to 80% B in 4.5 min and kept for 1 min, with a flow rate of 0.6 mL/min. The ESI-MS system operated at a 25 V cone voltage, a 600 °C probe temperature, and a 120 °C source temperature. ^1^H and ^13^C NMR spectra were measured on a JEOL ECA 400II NMR spectrometer (^1^H: 399.78 MHz, ^13^C: 100.53 MHz). Chemical shifts (δ) are reported in ppm and referenced to the middle peak of the solvent signal (CDCl_3_: 7.26 ppm, 77.00 ppm; DMSO-*d*_6_: 2.49 ppm, 39.5 ppm, explicitly indicated in the spectra, presented in the Supporting Information). High-resolution mass spectrometry (HRMS) measurements were performed on a UPLC Dionex Ultimate 3000 equipped with an Orbitrap Elite high-resolution mass spectrometer, Thermo Exactive Plus. The cross-coupling reactions under MW irradiation were performed in a 10 mL glass tube sealed with polytetrafluoroethylene-coated reusable septa. All MW reactions were carried out in a CEM-Discover MW reactor operating at 2.45 GHz with continuous irradiation of 300 W of maximum power. The MW irradiation of required power was used, and the temperature was continuously raised for 2 min. Once the desired temperature was reached, the mixture was held at this temperature for a given time. Thin layer chromatography (TLC) was performed on precoated silica gel 60 F254 plates and visualized by exposure to UV light (254 or 366 nm). Column chromatography was carried out by using silica gel grade 60, with mesh size of 230 to 400 Å. Melting points were measured on a Boetius stage apparatus and are uncorrected. Tested purines were dried at 120 °C for 45 min.

### General Procedure for the Analytical Study of *tert*-Butylation Reactions of Purines

To a suspension of the corresponding purine derivative (0.25 mmol) in anhydrous solvent (DCE or ACN; 2 mL) was added BSA (92 μL, 0.38 mmol) under argon. This mixture was heated at 76–80 °C (temperature of the oil bath) for 30 min to obtain a clear solution. After cooling in an ice bath, a Lewis acid (Table 1) was added. Next, the ice bath was removed, and stirring was continued at room temperature for 10 min. Then, *tert*-butyl bromide or other studied alkyl halide was added, and the mixture was stirred (amounts, temperatures, and durations are given in Table 1). The reaction mixture was then quenched with isopropyl alcohol (0.5 mL) and analyzed by the LC/MS method. In general, *N*^7^ isomers are more polar than *N*^9^ isomers and have lower retention times on a C-18 column, similarly on silica gel TLC plates.

#### 7-(*tert*-Butyl)-6-chloro-7*H*-purine (**2**)

To a suspension of 6-chloropurine (**1**) (775 mg, 5.0 mmol) in anhydrous DCE (40 mL) was added BSA (1.84 mL, 7.5 mmol) under argon. This mixture was heated at 76–80 °C (temperature of the oil bath) for 30 min to obtain a clear solution. After the mixture was cooled in an ice bath, SnCl_4_ (1.23 mL, 10.5 mmol) was added. Next, the ice bath was removed, and stirring was continued at room temperature for 10 min. Then, *tert*-butyl bromide (1.68 mL, 15 mmol) was added, and the mixture was stirred at room temperature for 19 h. Afterward, the reaction mixture was quenched with isopropyl alcohol (10 mL), diluted with chloroform (60 mL), and washed with water (40 mL), a saturated solution of NaHCO_3_ (2 × 60 mL), water (30 mL), and brine (60 mL). The organic phase was dried (MgSO_4_), and the solvents were evaporated under reduced pressure to obtain a yellowish crystalline compound as a crude product of sufficient purity. The yield was 792 mg (75%). A sample for analysis was obtained by crystallization from ethanol to obtain a white crystalline compound, mp: 183–185 °C (dec.). ^1^H NMR (400 MHz, CDCl_3_): δ 8.85 (s, 1H), 8.47 (s, 1H), 1.91 (s, 9H). ^13^C{^1^H} NMR (101 MHz, CDCl_3_): δ 163.9, 151.8, 146.9, 142.8, **123.2**, 59.1, 31.2. ^1^H NMR (400 MHz, DMSO-*d*_6_): δ 8.88 (s, 1H, H-8), 8.79 (s, 1H, H-2), 1.83 (s, 9H, (CH_3_)_3_). ^13^C{^1^H} NMR (101 MHz, DMSO-*d*_6_): δ 163.5 (C-4), 151.1 (C-2), 149.1 (C-8), 141.7 (C-6), **122.6 (C-5)**, 58.8 (C), 30.4 (CH_3_). HRMS (ESI, *m*/*z*): [M + H]^+^ calcd for C_9_H_12_ClN_4_, 211.0745; found, 211.0746.

With the mentioned method in DCE, 5 g of chloropurine was processed to obtain 4.7 g (69%) of crude compound **2** (98% purity based on ^1^H NMR).

The title compound **2** was prepared in a similar manner using anhydrous ACN (40 mL) instead of DCE. In this way, the reaction time was shortened to 3 h at room temperature after the addition of *tert*-butyl bromide. The yield was 825 mg (78%) as a crude product of sufficient purity (97% purity based on ^1^H NMR).

#### 9-(*tert*-Butyl)-6-chloro-9*H*-purine (**3**)

This compound was prepared in a similar manner as purine **2** using 6-chloropurine (155 mg, 1 mmol), BSA (368 μL, 1.5 mmol), SnCl_4_ (248 μL, 2.1 mmol), and *tert*-butyl bromide (336 μL, 3 mmol) in ACN (8 mL). This reaction was not carried out at room temperature but at 80 °C for 5 h after the addition of *tert*-butyl bromide. After processing the reaction mixture and evaporating the solvents, the crude product (120 mg) was crystallized twice from isopropanol–water (1:1, v/v) to give a pale-yellow crystalline compound. The yield was 82 mg (39%), mp: 144–146 °C (lit,^51^ 144–146 °C). ^1^H NMR (400 MHz, CDCl_3_): δ 8.72 (s, 1H), 8.20 (s, 1H), 1.83 (s, 9H). ^13^C{^1^H} NMR (101 MHz, CDCl_3_): δ 152.0, 151.1, 150.9, 142.9, **132.7**, 58.5, 28.9. ^1^H NMR (400 MHz, DMSO-*d*_6_): δ 8.76 (s, 1H, H-2), 8.68 (s, 1H, H-8), and 1.75 (s, 9H, (CH_3_)_3_). ^13^C{^1^H} NMR (101 MHz, DMSO-*d*_6_): δ 151.8 (C-4), 150.5 (C-2), 149.2 (C-6), 145.4 (C-8), **132.0 (C-5)**, 58.2 (C), 28.3 (CH_3_). HRMS (ESI, *m*/*z*): [M + H]^+^ calcd for C_9_H_12_ClN_4_, 211.0745; found, 211.0746.

#### 7-(*tert*-Pentyl)-6-chloro-7*H*-purine (**4**)

This compound was prepared in a similar manner as purine **2** using 6-chloropurine (155 mg, 1 mmol), BSA (368 μL, 1.5 mmol), SnCl_4_ (248 μL, 2.1 mmol), and 2-bromo-2-methylbutane (383 μL, 3 mmol) in DCE (8 mL). The yield was 119 mg (53%) as a crude product. A sample for analysis was obtained by the crystallization form ethanol to obtain a white crystalline compound, mp: 95–100 °C. ^1^H NMR (400 MHz, CDCl_3_): δ 8.84 (s, 1H), 8.42 (s, 1H), 2.30 (q, *J* = 7.5 Hz, 2H), 1.85 (s, 6H), 0.77 (t, *J* = 7.5 Hz, 3H). ^13^C{^1^H} NMR (101 MHz, CDCl3): δ 163.9, 151.7, 147.6, 142.9, **123.2**, 62.3, 34.5, 28.7, 8.4. HRMS (ESI, *m*/*z*): [M + H]^+^ calcd for C_10_H_14_ClN_4_, 225.0902; found, 225.0897.

#### 9-(*tert*-Pentyl)-6-chloro-9*H*-purine (**5**)

This compound was prepared in a similar manner as purine **2** using 6-chloropurine (309 mg, 2 mmol), BSA (736 μL, 3 mmol), SnCl_4_ (496 μL, 4.2 mmol), and 2-bromo-2-methylbutane (766 μL, 6 mmol) in ACN (16 mL). This reaction was carried out not at room temperature but at 80 °C for 3 h after the addition of 2-bromo-2-methylbutane. After processing the reaction mixture and evaporating the solvents, the crude product was dried under high vacuum (10^–2^ Torr) for 1 h to obtain an impure product (280 mg) which was crystallized twice from isopropanol–water (1:1, v/v) to give a pale-yellow crystalline compound. The yield was 160 mg (36%), mp: 103–105 °C. ^1^H NMR (400 MHz, CDCl_3_): δ 8.70 (s, 1H), 8.14 (s, 1H), 2.24 (q, *J* = 7.4 Hz, 2H), 1.79 (s, 6H), 0.70 (t, *J* = 7.5 Hz, 3H). ^13^C{^1^H} NMR (101 MHz, CDCl_3_): δ 151.9, 151.0, 150.8, 143.6, **132.6**, 61.6, 32.7, 26.6, 8.1. HRMS (ESI, *m*/*z*): [M + H]^+^ calcd for C_10_H_14_ClN_4_, 225.0902; found, 225.0897.

#### 7-(*tert*-Butyl)-6-chloro-2-(methylthio)-7*H*-purine (**7**)

This compound was prepared in a similar manner as purine **2** using 6-chloro-2-methylthiopurine (**6**) (200 mg, 1 mmol), BSA (368 μL, 1.5 mmol), SnCl_4_ (248 μL, 2.1 mmol), and *tert*-butyl bromide (672 μL, 6 mmol) in ACN (8 mL). This reaction was carried out at room temperature for 4 h. After processing the reaction mixture and evaporating the solvents, the crude product (170 mg) was crystallized from isopropanol to obtain a white crystalline compound. The yield was 133 mg (52%), mp: 177–179 °C (dec). ^1^H NMR (400 MHz, CDCl_3_): δ 8.33 (s, 1H), 2.64 (s, 3H), 1.87 (s, 9H). ^13^C{^1^H} NMR (101 MHz, CDCl_3_): δ 165.7, 164.8, 146.5, 142.7, 120.0, 58.9, 31.0, 14.4. HRMS (ESI, *m*/*z*): [M + H]^+^ calcd for C_10_H_14_ClN_4_S, 257.0622; found, 257.0625.

#### 9-(*tert*-Butyl)-6-chloro-2-(methylthio)-9*H*-purine (**8**)

This compound was prepared in a similar manner as purine **2** using 6-chloro-2-methylthiopurine (**6**) (200 mg, 1 mmol), BSA (368 μL, 1.5 mmol), SnCl_4_ (248 μL, 2.1 mmol), and *tert*-butyl bromide (672 μL, 6 mmol) in ACN (8 mL). This reaction was carried out not at room temperature but at 80 °C for 5 h after the addition of *tert*-butyl bromide. After processing the reaction mixture and evaporating the solvents, the crude product (120 mg) was purified by column chromatography (3 cm ID, silica gel, DCM–MeOH, 40:1, v/v) and crystallized from isopropanol–water (1:1, v/v) to obtain a white crystalline compound. The yield was 72 mg (28%), mp: 144–146 °C. ^1^H NMR (400 MHz, CDCl_3_): δ 8.03 (s, 1H), 2.62 (s, 3H), 1.81 (s, 9H). ^13^C{^1^H} NMR (101 MHz, CDCl_3_): δ: 165.1, 152.7, 150.9, 141.5, 129.6, 58.2, 28.9, 14.8. HRMS (ESI, *m*/*z*): [M + H]^+^ calcd for C_10_H_14_ClN_4_S, 257.0622; found, 257.0621.

#### 7-(*tert*-Butyl)-6-methoxy-7*H*-purine (**11**)

##### Method A

To a mixture of 7-(*tert*-butyl)-6-chloropurine (**2**) (150 mg, 0.71 mmol) in anhydrous methanol (3 mL) was added a solution of sodium methoxide in methanol (2.3 mL, 1 M MeONa). This mixture was stirred for 30 min at room temperature and then neutralized with dilute acetic acid, followed by the addition of toluene (8 mL). The organic phase was washed with brine (2 × 4 mL), dried (MgSO_4_), and evaporated under reduced pressure to obtain a white crystalline solid. The yield was 97 mg (66%) as a crude product. ^1^H NMR (400 MHz, CDCl_3_): δ 8.59 (s, 1H, H-2), 8.15 (s, 1H, H-8), 4.15 (s, 3H), 1.71 (s, 9H). ^13^C{^1^H} NMR (101 MHz, CDCl_3_): δ 163.2, 155.8, 151.7, 143.2, 112.5, 57.9, 54.0, 30.1. HRMS (ESI, *m*/*z*): [M + H]^+^ calcd for C_10_H_15_N_4_O, 207.1240; found, 207.1243.

##### Method B

The title compound **11** was prepared in a similar manner as purine **2** using 6-methoxypurine (**9**) (150 mg, 1 mmol), BSA (368 μL, 1.5 mmol), SnCl_4_ (248 μL, 2.1 mmol), and *tert*-butyl bromide (336 μL, 3 mmol) in ACN (8 mL). The reaction time was 5 h at room temperature. The yield was 124 mg (60%) as a crude product. A sample for analysis was obtained by crystallization from hexane–EtOAc (5:2, v/v) to obtain a white crystalline compound, mp: 117–119 °C. This method gave product **11** with the same spectral characteristics as Method A.

#### 7-(*tert*-Butyl)-6-(methylthio)-7*H*-purine (**12**)

This compound was prepared in a similar manner as purine **2** using 6-(methylthio)purine (**10**) (166 mg, 1 mmol), BSA (368 μL, 1.5 mmol), SnCl_4_ (248 μL, 2.1 mmol), and *tert*-butyl bromide (336 μL, 3 mmol) in ACN (8 mL). The reaction time was 3 h at room temperature. The yield was 150 mg (68%) as a crude product. A sample for analysis was obtained by crystallization form hexane–EtOAc (5:2, v/v) to obtain a white crystalline compound, mp: 134–136 °C. ^1^H NMR (400 MHz, CDCl_3_): δ 8.80 (s, 1H, H-2), 8.27 (s, 1H, H-8), 2.71 (s, 3H), 1.85 (s, 9H). ^13^C{^1^H} NMR (101 MHz, CDCl_3_): δ 160.2, 153.3, 151.3, 144.6, 123.9, 57.9, 31.6, 14.4. HRMS (ESI, *m*/*z*): [M + H]^+^ calcd for C_10_H_15_N_4_S, 223.1012; found, 223.1008.

#### 7-(*tert*-Butyl)-7*H*-purin-6-ol (**13**)

To a mixture of 7-(*tert*-butyl)-6-chloropurine (**2**) (100 mg, 0.48 mmol) in water (4 mL) was added sodium hydroxide (60 mg, 1.5 mmol). The resulting solution was heated in a sealed vial for 30 min at 100 °C and then cooled and neutralized with dilute acetic acid. After the mixture was left to stand overnight at 2 °C, the precipitated white crystalline compound was filtered off, washed with water, and dried. The yield was 60 mg (66%), mp: 268–272 °C (dec). ^1^H NMR (400 MHz, CDCl_3_): δ 11.62 (bs, 1H), 8.16 (s, 1H), 8.10 (s, 1H), 1.82 (s, 9H). ^13^C{^1^H} NMR (101 MHz, CDCl_3_): δ 159.9, 155.3, 144.5, 141.2, 115.6, 58.6, 29.9. HRMS (ESI, *m*/*z*): [M + H]^+^ calcd for C_9_H_13_N_4_O_1_, 193.1084; found, 193.1085.

#### 7-(*tert*-Butyl)-6-ethoxy-7*H*-purine (**14**)

To a mixture of 7-(*tert*-butyl)-6-chloropurine (**2**) (100 mg, 0.48 mmol) in anhydrous ethanol (2 mL) was added a solution of sodium ethoxide in ethanol (1.5 mL, 1 M EtONa). This mixture was stirred for 30 min at room temperature, neutralized with dilute acetic acid, and toluene (5 mL) was then added. The organic phase was washed with brine (2 × 3 mL), dried (MgSO_4_), and evaporated under reduced pressure to obtain a white crystalline solid. The yield was 98 mg (93%) as a crude product. A sample for analysis was obtained by crystallization form hexane–EtOAc (5:2, v/v) to obtain a white crystalline compound, mp: 85–87 °C. ^1^H NMR (400 MHz, CDCl_3_): δ 8.60 (s, 1H), 8.18 (s, 1H), 4.66 (q, *J* = 7.2 Hz, 2H), 1.76 (s, 9H), 1.51 (t, *J* = 7.2 Hz, 3H). ^13^C{^1^H} NMR (101 MHz, CDCl_3_): δ 163.3, 155.6, 151.8, 143.2, 112.6, 63.1, 58.0, 30.2, 14.4. HRMS (ESI, *m*/*z*): [M + H]^+^ calcd for C_11_H_17_N_4_O, 221.1397; found, 221.1398.

#### 7-(*tert*-Butyl)-1,7-dihydro-6*H*-purin-6-thione (**15**)

To a solution of 7-(*tert*-butyl)-6-chloropurine (**2**) (150 mg, 0.71 mmol) in DMF (4.3 mL) was added a solution of NaSH in water (893 μL, 4 M NaSH) under argon. The resulting mixture was stirred for 90 min at room temperature, then neutralized with dilute acetic acid and EtOAc (80 mL), and bubbled with argon. The organic phase was washed with brine (3 × 30 mL), dried (MgSO_4_), and evaporated under reduced pressure to obtain a white solid as a crude product. The yield was 104 mg (70%). ^1^H NMR (400 MHz, DMSO-*d*_6_): δ 13.60 (bs, 1H), 8.58 (s, 1H), 8.15 (d, *J* = 4.0 Hz, 1H), 1.92 (s, 9H). ^13^C{^1^H} NMR (101 MHz, DMSO-*d*_6_): δ 169.9, 156.1, 146.1, 144.8, 127.3, 59.5, 30.8. HRMS (ESI, *m*/*z*): [M + H]^+^ calcd for C_9_H_13_N_4_S, 209.0855; found, 209.0854.

#### 6-Azido-7-(*tert*-butyl)-7*H*-purine (**16**)

To a solution of 7-(*tert*-butyl)-6-chloropurine (**2**) (300 mg, 1.43 mmol) in DMSO (5.7 mL) was added sodium azide (279 mg, 4.3 mmol). The resulting mixture was heated for 4 h at 60 °C, diluted with EtOAc (100 mL), and the organic layer was washed with brine (3 × 40 mL). Then, the organic phase was dried (MgSO_4_) and concentrated under reduced pressure to obtain a white crystalline compound as a crude product. The yield was 282 mg (91%). ^1^H NMR (400 MHz, CDCl_3_): δ 9.57 (s, 1H), 8.34 (s, 1H), 1.97 (s, 9H). ^13^C{^1^H} NMR (101 MHz, CDCl_3_): δ 152.8, 143.2, 142.0, 133.6, 110.7, 59.4, 29.2. HRMS (ESI, *m*/*z*): [M + H]^+^ calcd for C_9_H_12_N_7_, 218.1149; found, 218.1149.

#### 7-(*tert*-Butyl)-7*H*-purin-6-amine (**17**)

To a solution of azide **16** (100 mg, 0.46 mmol) in ethanol (12 mL) was added palladium on charcoal (25 mg, 10% Pd/C). This mixture was hydrogenated under a hydrogen pressure of 5 bar for 24 h, filtered through a syringe microfilter (0.22 μm, 2.5 cm ID), and evaporated under reduced pressure. The residue was crystallized from water (about 2 mL) to obtain a white crystalline compound. The yield was 66 mg (75%), mp: 287–290 °C. ^1^H NMR (400 MHz, DMSO-*d*_6_): δ:8.35 (s, 1H), 8.20 (s, 1H), 6.67 (s, 2H), 1.69 (s, 9H). ^13^C{^1^H} NMR (101 MHz, DMSO-*d*_6_): δ 161.5, 151.8, 150.7, 143.5, 110.7, 56.2, 30.9. HRMS (ESI, *m*/*z*): [M + H]^+^ calcd for C_9_H_14_N_5_, 192.1244; found, 192.1243.

#### *N*-Benzyl-7-(*tert*-butyl)-7*H*-purin-6-amine (**18**)

To a solution of 7-(*tert*-butyl)-6-chloropurine (**2**) (105 mg, 0.5 mmol) in butanol (2 mL) were added benzylamine (81 μL, 0.75 mmol) and DIPEA (174 μL, 1 mmol). The reaction mixture was heated for 6.5 h at 120 °C. After evaporation of the volatiles under reduced pressure, the residue was purified by column chromatography (4 cm ID) on silica gel using DCM–methanol (80:1, v/v) to obtain a light orange colored compound. The yield was 90 mg (64%). ^1^H NMR (400 MHz, CDCl_3_): δ 8.57 (s, 1H), 8.12 (s, 1H), 7.30–7.40 (m, 5H), 5.34 (s, 1H), 4.88 (d, *J* = 5.2 Hz, 2H), 1.75 (s, 9H). ^13^C{^1^H} NMR (101 MHz, CDCl_3_): δ 161.6, 152.8, 149.5, 142.1, 138.3, 128.9, 127.70, 127.68, 111.8, 56.1, 45.9, 31.7. HRMS (ESI, *m*/*z*): [M + H]^+^ calcd for C_16_H_20_N_5_, 282.1713; found, 282.1711.

#### 7-(*tert*-Butyl)-*N*-cyclohexyl-7*H*-purin-6-amine (**19**)

To a solution of 7-(*tert*-butyl)-6-chloropurine (**2**) (110 mg, 0.52 mmol) in butanol (2 mL) were added cyclohexylamine (120 μL, 1.04 mmol) and DIPEA (182 μL, 1.02 mmol). The reaction mixture was heated for 13 h at 120 °C. After that time, additional cyclohexylamine (60 μL) was added to increase the conversion, and heating was extended for another 2 h. Next, the volatiles were evaporated under reduced pressure, and the residue was purified by column chromatography (4 cm ID) on silica gel using DCM–methanol (80:3, v/v) to obtain a yellowish colored compound. The yield was 108 mg (76%). ^1^H NMR (400 MHz, CDCl_3_): δ 8.45 (s, 1H), 8.04 (s, 1H), 4.99 (d, *J* = 7.0 Hz, 1H), 4.20–4.29 (m, 1H), 2.07–2.11 (m, 2H), 1.75 (s, 9H), 1.58–1.72 (m, 3H), 1.41–1.51 (m, 2H), 1.19–1.32 (m, 3H). ^13^C{^1^H} NMR (101 MHz, CDCl_3_): δ 161.2, 152.7, 149.1, 141.7, 111.6, 55.9, 49.5, 33.0, 31.7, 25.5, 24.5. HRMS (ESI, *m*/*z*): [M + H]^+^ calcd for C_15_H_24_N_5_, 274.2026; found, 274.2028.

#### 3-(((7-(*tert*-Butyl)-7*H*-purin-6-yl)amino)methyl)phenol (**20**)

This compound was prepared in a similar manner as adenine **18** using 7-(*tert*-butyl)-6-chloropurine (**2**) (110 mg, 0.52 mmol), 3-hydroxybenzylamine (128 mg, 1.04 mmol), and DIPEA (182 μL, 1.04 mmol) in butanol (2 mL). The reaction time was 3 h at 120 °C; mobile phase: DCM–methanol (80:3, v/v) for column separation. The yield was 73 mg (47%) as a light yellow crystalline compound. ^1^H NMR (400 MHz, CDCl_3_): δ 9.79 (bs, 1H), 8.40 (s, 1H), 8.03 (s, 1H), 7.18 (t, *J* = 7.8 Hz, 1H), 7.10 (t, *J* = 1.8 Hz, 1H), 6.89 (dd, *J* = 8.1, 2.3 Hz, 1H), 6.81 (d, *J* = 7.6 Hz, 1H), 5.48 (t, *J* = 5.2 Hz, 1H), 4.81 (d, *J* = 5.2 Hz, 2H), 1.71 (s, 9H). ^13^C{^1^H} NMR (101 MHz, CDCl_3_): δ 160.4, 158.0, 152.3, 149.8, 142.0, 139.5, 129.9, 118.5, 115.3, 114.8, 111.7, 56.5, 45.7, 31.7. HRMS (ESI, *m*/*z*): [M + H]^+^ calcd for C_16_H_20_N_5_O, 298.1662; found, 298.1662.

#### 7-(*tert*-Butyl)-*N*-(furan-2-ylmethyl)-7*H*-purin-6-amine (**21**)

This compound was prepared in a similar manner as adenine **18** using 7-(*tert*-butyl)-6-chloropurine (**2**) (110 mg, 0.52 mmol), furfurylamine (100 μL, 1.04 mmol), and DIPEA (182 μL, 1.04 mmol) in butanol (2 mL). The reaction time was 3 h at 120 °C; mobile phase: DCM–methanol (40:2, v/v) for column separation. The yield was 106 mg (75%) as a light yellow crystalline compound. ^1^H NMR (400 MHz, CDCl_3_): δ 8.56 (s, 1H), 8.11 (s, 1H), 7.37 (d, *J* = 0.8 Hz, 1H), 6.33–6.34 (m, 1H), 6.30 (d, *J* = 4.0 Hz, 1H), 5.44 (bs, 1H), 4.86 (d, *J* = 5.2 Hz, 2H), 1.77 (s, 9H). ^13^C{^1^H} NMR (101 MHz, CDCl_3_): δ 161.6, 152.6, 151.3, 149.2, 142.3, 142.2, 112.0, 110.6, 107.5, 56.2, 38.7, 31.6. HRMS (ESI, *m*/*z*): [M + H]^+^ calcd for C_14_H_18_N_5_O, 272.1506; found, 272.1505.

#### 7-(*tert*-Butyl)-6-(piperidin-1-yl)-7*H*-purin (**22**)

This compound was prepared in a similar manner as adenine **18** using 7-(*tert*-butyl)-6-chloropurine (**2**) (110 mg, 0.52 mmol), piperidine (256 μL, 2.6 mmol), and DIPEA (182 μL, 1.04 mmol) in butanol (2 mL). The reaction time was 3 h at 120 °C. The reaction mixture was worked up by dilution with toluene (5 mL) and extraction with brine (2 × 8 mL). The organic phase was dried (MgSO_4_) and evaporated under reduced pressure to obtain a light orange crystalline compound. The yield was 118 mg (87%) A sample for analysis was obtained by crystallization form ethanol–water, mp: 167–169 °C. ^1^H NMR (400 MHz, CDCl_3_): δ 8.89 (s, 1H), 8.33 (s, 1H), 3.04 (t, *J* = 5.3 Hz, 4H), 1.83 (s, 9H), 1.71–1.76 (m, 4H), 1.65–1.67 (m, 2H). ^13^C{^1^H} NMR (101 MHz, CDCl_3_): δ 164.8, 158.7, 152.7, 146.3, 119.6, 59.0, 53.1, 30.0, 25.6, 23.8. HRMS (ESI, *m*/*z*): [M + H]^+^ calcd for C_14_H_22_N_5_, 260.1870; found, 260.1873.

#### 7-(*tert*-Butyl)-6-(4-methoxyphenyl)-7*H*-purine (**23**)

A MW vial was charged with a magnetic stirring bar, 7-(*tert*-butyl)-6-chloropurine (**2**) (100 mg, 0.48 mmol), 4-methoxyphenylboronic acid (109 mg, 0.72 mmol), K_2_CO_3_ (83 mg, 0.6 mmol), Pd(PPh_3_)_4_ (26.7 mg, 0.023 mmol), and toluene (6 mL). The vial was sealed and inserted into a MW reactor, where it was irradiated at variable power, so the temperature was maintained at 140 °C for 30 min. After that time, the volatiles were removed under reduced pressure, and the residue was purified by column chromatography (4 cm ID, silica gel, DCM–MeOH, 80:3, v/v) to yield the title product as a white crystalline compound. The yield was 88 mg (66%). ^1^H NMR (400 MHz, CDCl_3_): δ 9.05 (s, 1H), 8.44 (s, 1H), 7.37 (d, *J* = 8.5 Hz, 2H), 7.00 (d, *J* = 8.5 Hz, 2H), 3.87 (s, 3H), 1.47 (s, 9H). ^13^C{^1^H} NMR (101 MHz, CDCl_3_): δ 162.8, 160.3, 153.2, 151.7, 147.5, 132.6, 130.3, 124.1, 113.8, 58.7, 55.4, 30.8. HRMS (ESI, *m*/*z*): [M + H]^+^ calcd for C_16_H_19_N_4_O, 283.1553; found, 283.1551.

#### 7-(*tert*-Butyl)-6-(thiophen-3-yl)-7*H*-purine (**24**)

This compound was prepared in a similar manner as purine derivative **23** using 7-(*tert*-butyl)-6-chloropurine (**2**) (100 mg, 0.48 mmol), 3-thienylboronic acid (92 mg, 0.72 mmol), K_2_CO_3_ (83 mg, 0.6 mmol), Pd(PPh_3_)_4_ (26.7 mg, 0.023 mmol), and toluene (6 mL). The yield was 103 mg (84%), as a light orange colored compound. ^1^H NMR (400 MHz, CDCl_3_): δ 9.06 (s, 1H), 8.45 (s, 1H), 7.47 (dd, *J* = 8.1, 3.2 Hz, 2H), 7.24 (d, *J* = 4.6 Hz, 1H), 1.50 (s, 9H). ^13^C{^1^H} NMR (101 MHz, CDCl_3_): δ 162.9, 151.7, 149.0, 147.5, 140.1, 128.6, 126.5, 125.9, 124.5, 58.6, 30.6. HRMS (ESI, *m*/*z*): [M + H]^+^ calcd for C_13_H_15_N_4_S, 259.1012; found, 259.1012.

## Supporting Information Available

Copies of ^1^H and ^13^C NMR spectra (PDF)
