# Supplementary material for: Study of Direct N7 Regioselective tert-Alkylation of 6-Substituted Purines and Their Modification at Position C6 through O, S, N, and C Substituents
Source: ACS Omega. 2024 Apr 6;9(15):17368–78. doi: 10.1021/acsomega.4c00068 (PMC11024948; doi:10.1021/acsomega.4c00068)

Supporting Information

Study of Direct N^7 Regioselective *tert*-Alkylation of 6-Substituted Purines and Their Modification at Position C^6 through O, S, N and C Substituents

Filip Nevrlka, Adam Bědroň, Michal Valenta, Lenka Tranová, and Jakub Stýskala*

Department of Organic Chemistry, Faculty of Science, Palacký University, 17. Listopadu 12, 771 46 Olomouc, Czech Republic

*E-mail: jakub.styskala@upol.cz

Contents:

^{1}H and ^{13}C NMR data of prepared compounds **2-24**	Pages: S2-S23
NOESY NMR data of compounds **11** and **12**	Pages: S24-S25
HPLC chromatograms of Table 1, entry 18 and 25	Page: S26

^{1}H NMR (400 MHz, $CDCl_3$) 7-(*tert*-butyl)-6-chloro-7*H*-purine (**2**)

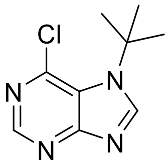

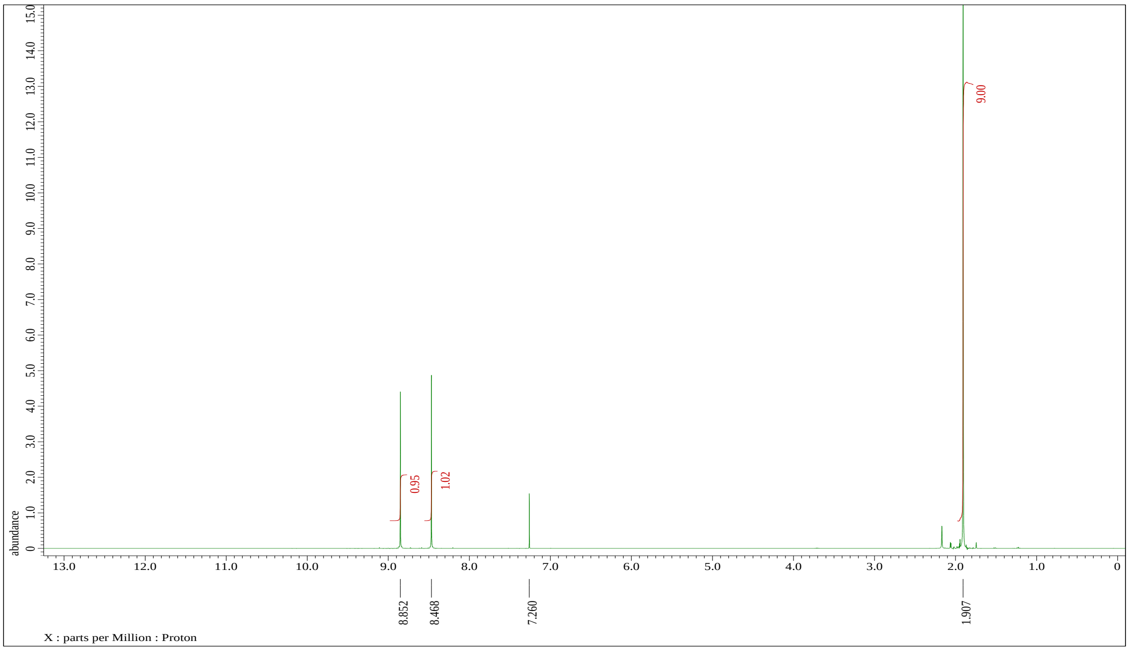

^{13}C{^{1}H} NMR (101 MHz, $CDCl_3$)

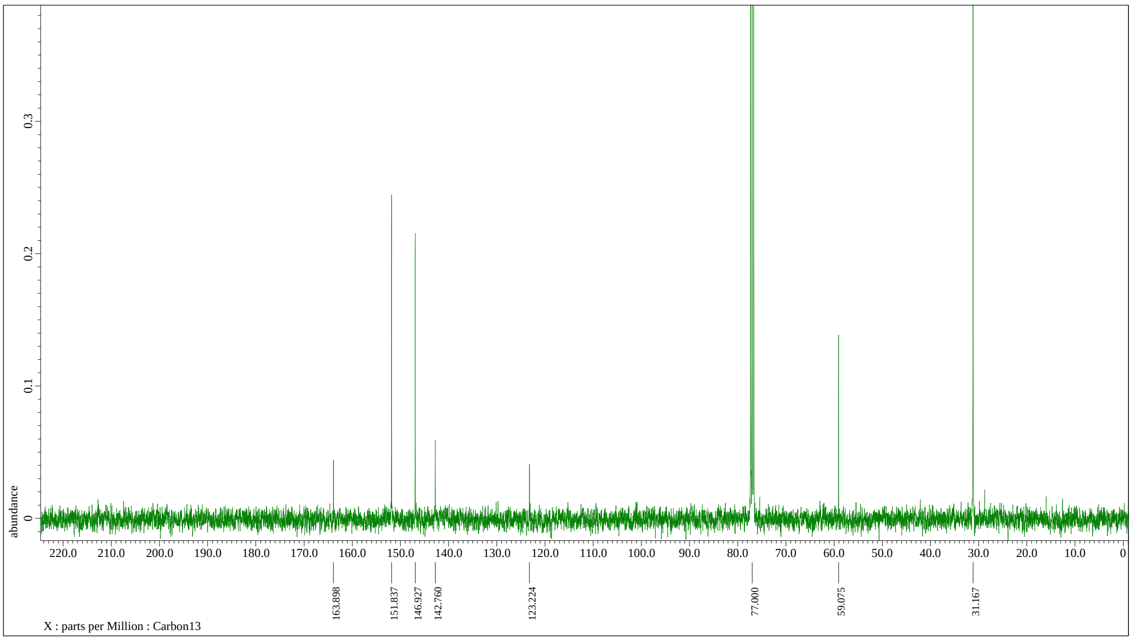

^{1}H NMR (400 MHz, DMSO-d_6) 7-(*tert*-butyl)-6-chloro-7*H*-purine (**2**)

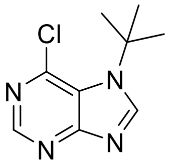

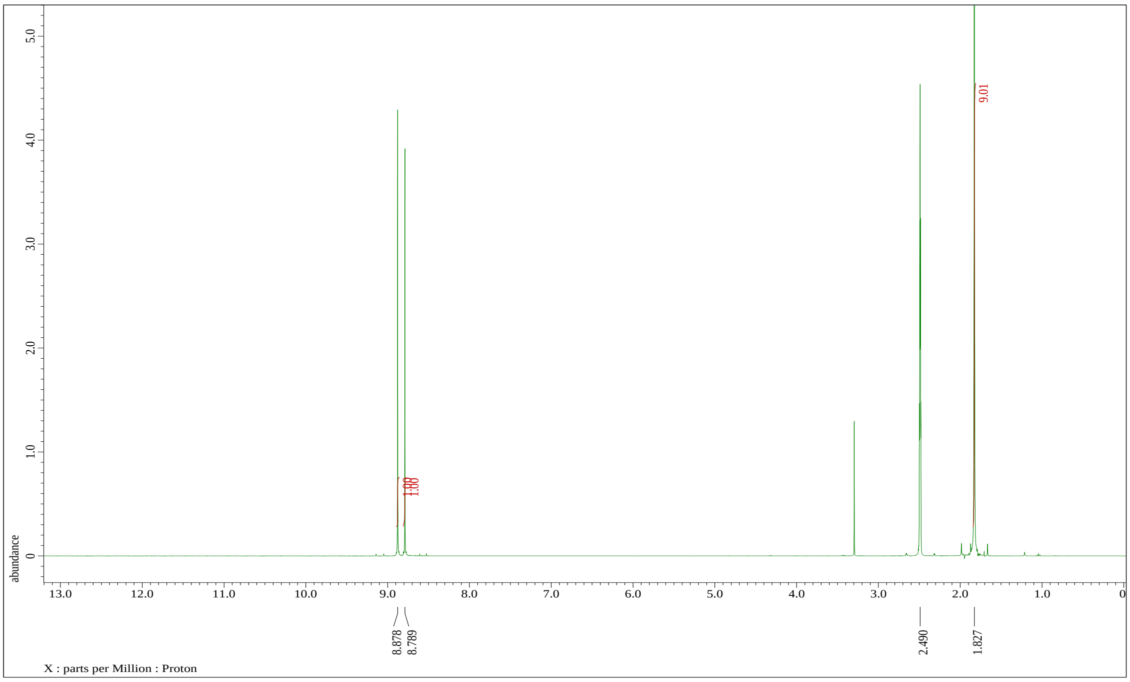

^{13}C{^{1}H} NMR (101 MHz, DMSO-d_6)

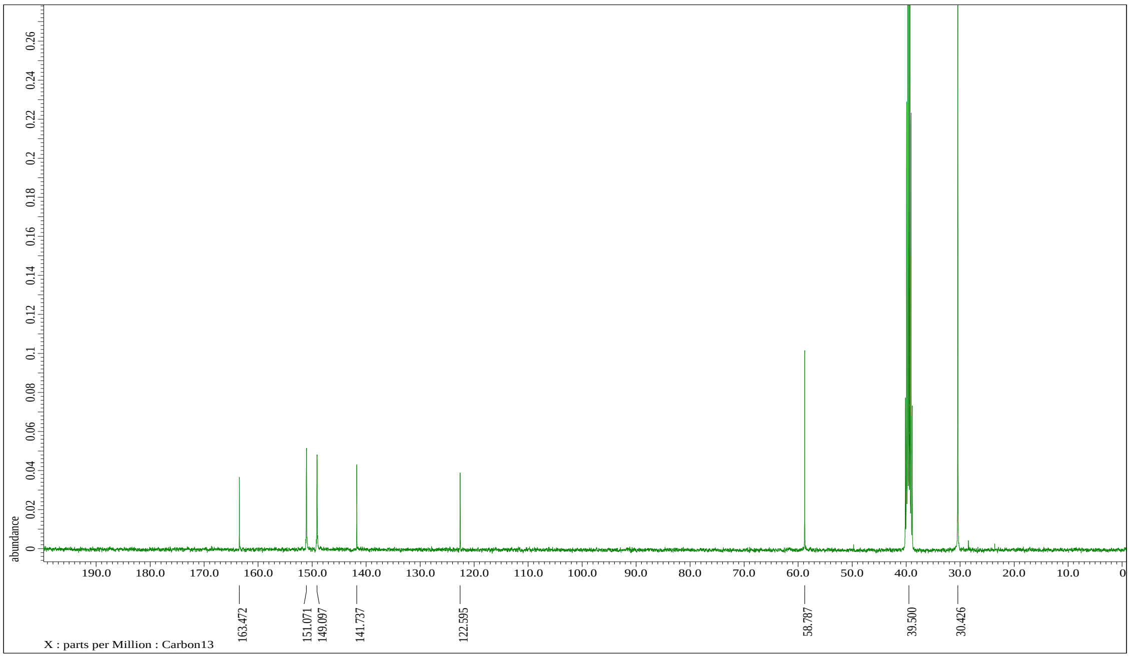

^{1}H NMR (400 MHz, $CDCl_3$) **9**-(*tert*-butyl)-6-chloro-9*H*-purine (**3**)

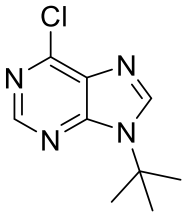

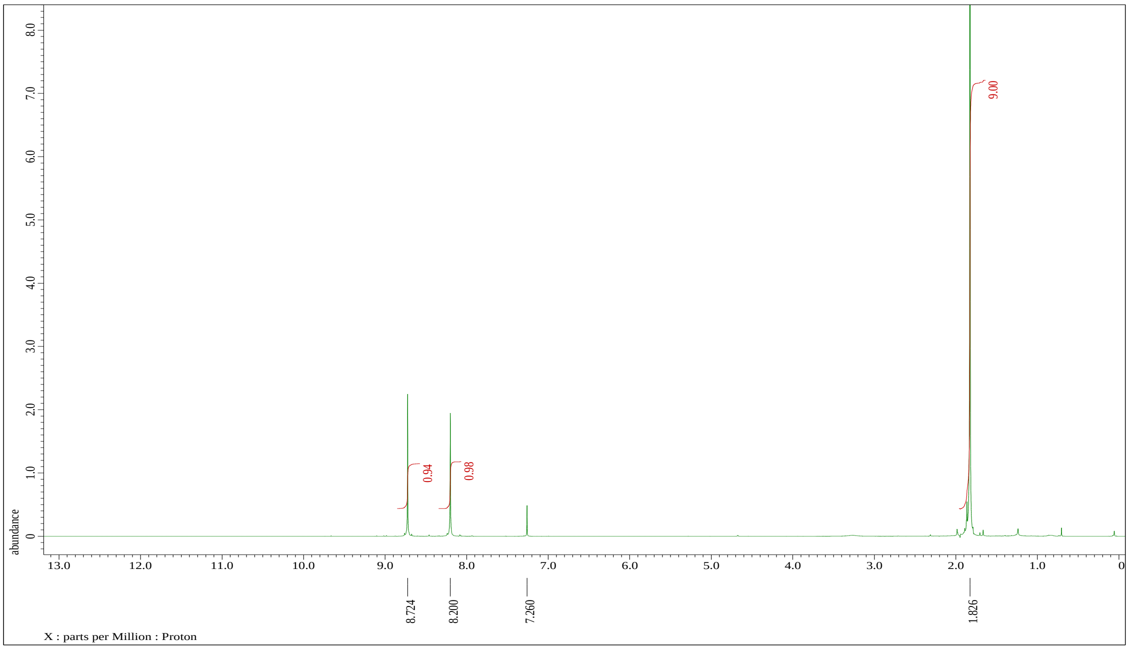

^{13}C NMR (101 MHz, $CDCl_3$)

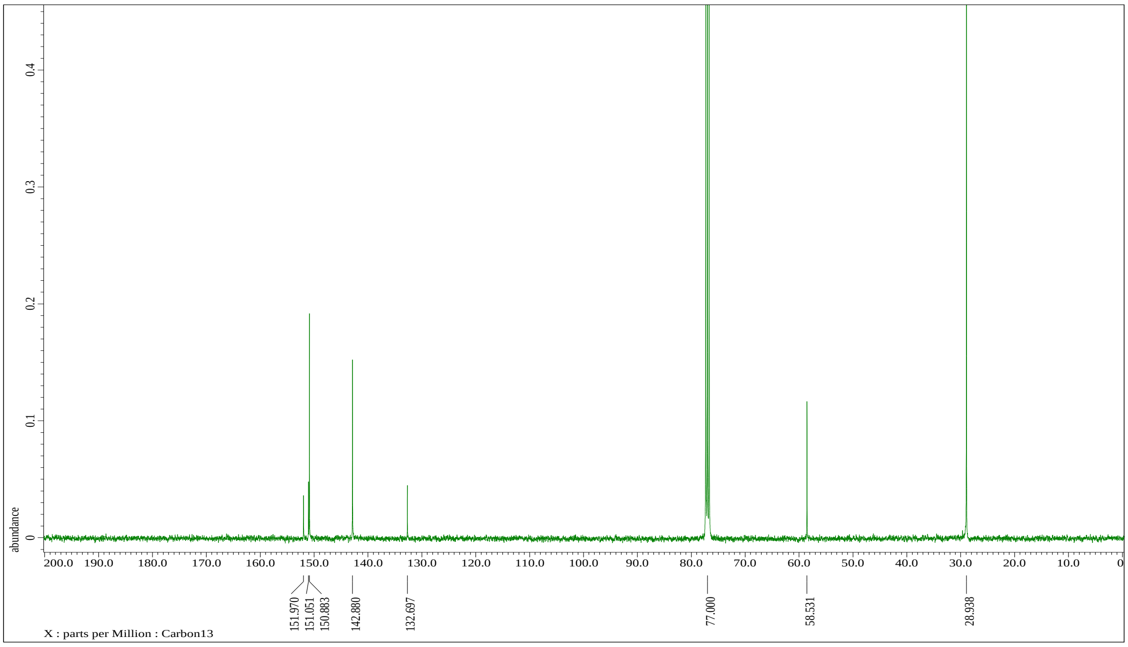

^{1}H NMR (400 MHz, DMSO-d_6) **9**-(*tert*-butyl)-6-chloro-9*H*-purine (**9**)

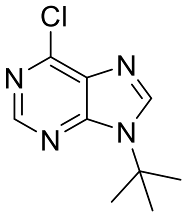

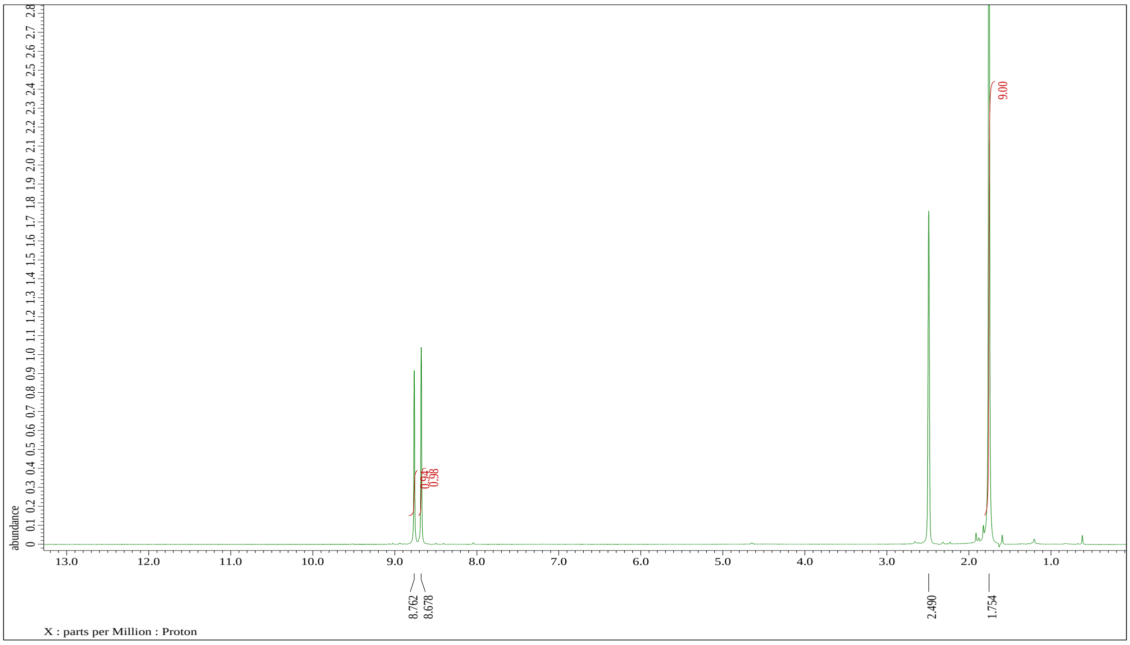

^{13}C NMR (101 MHz, DMSO-d_6)

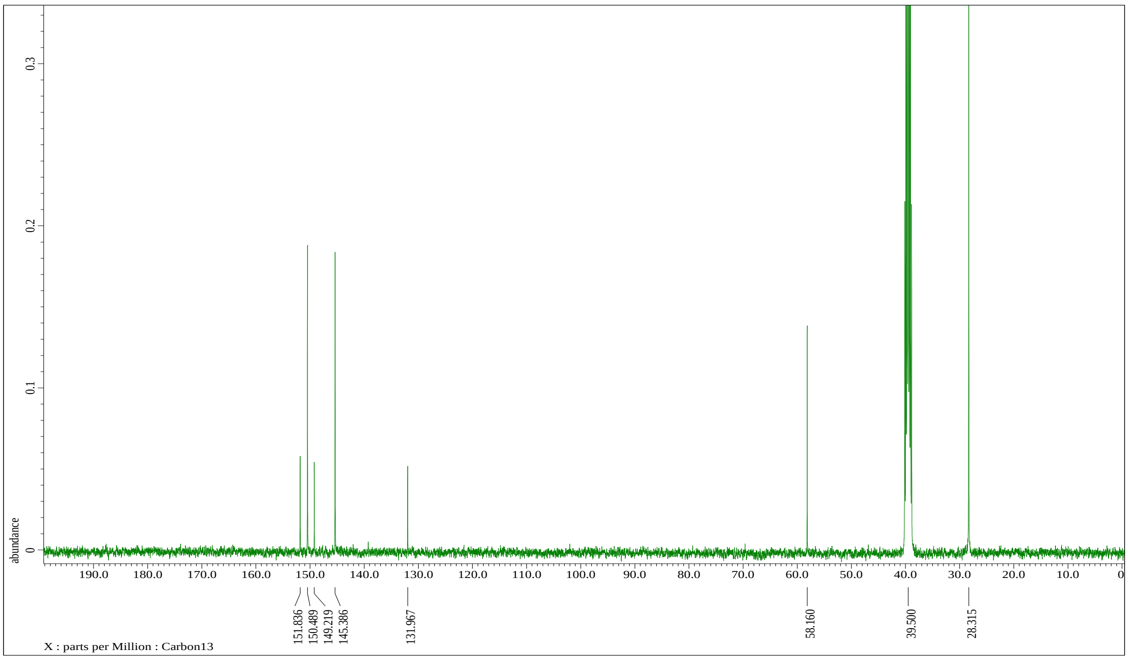

^{1}H NMR (400 MHz, $CDCl_3$) 6-chloro-7-(*tert*-pentyl)-7*H*-purine (**4**)

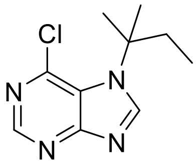

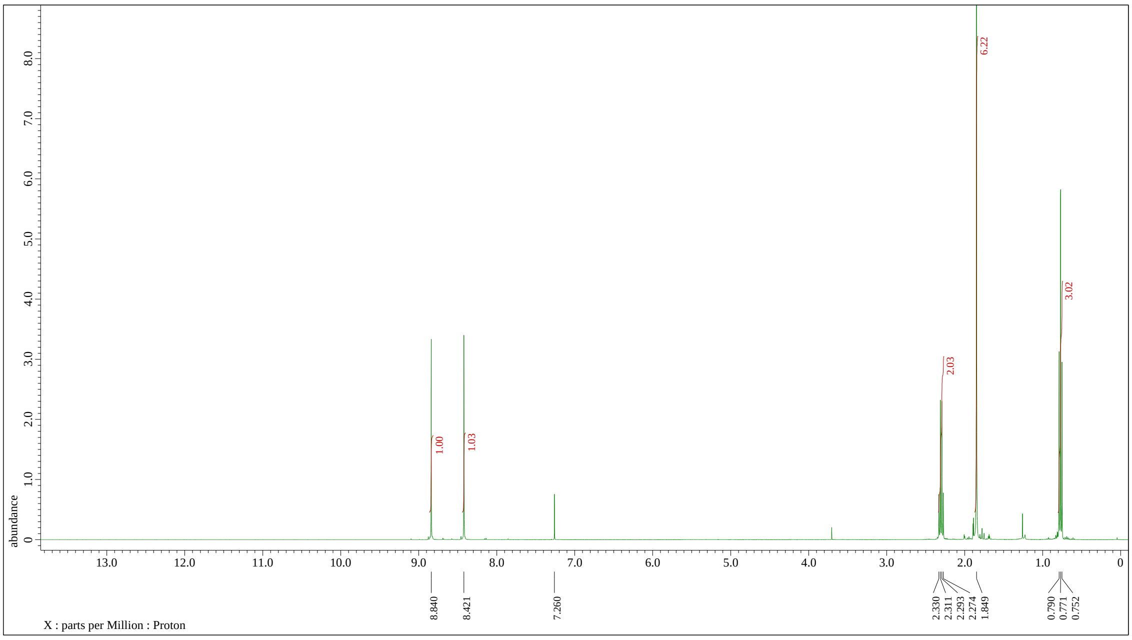

^{13}C{^{1}H} NMR (101 MHz, $CDCl_3$)

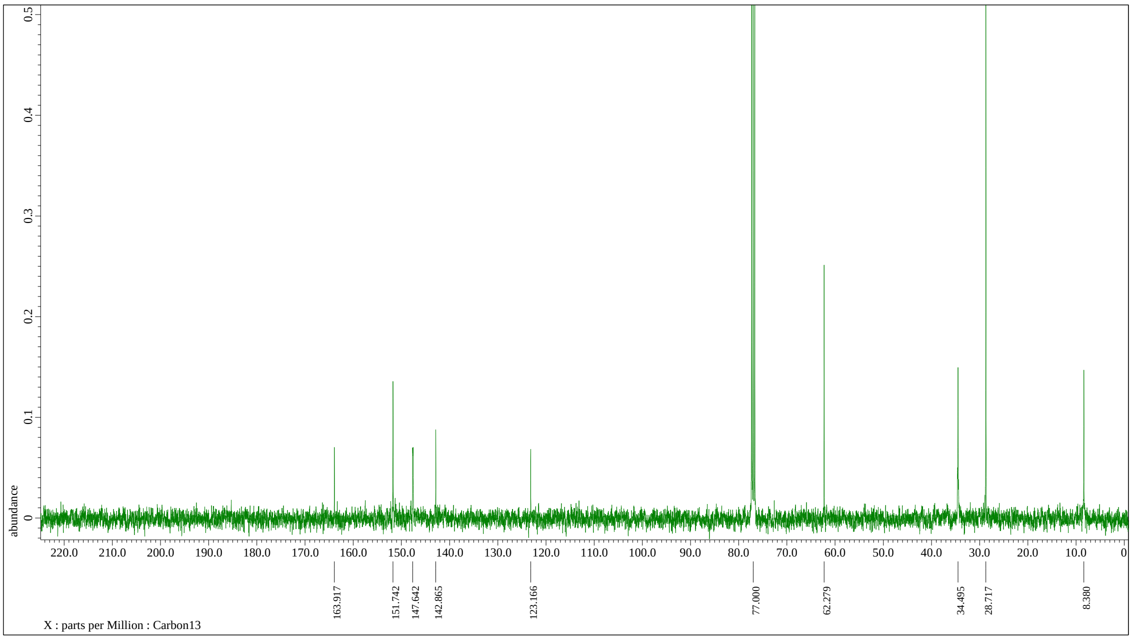

^{1}H NMR (400 MHz, $CDCl_3$) 6-chloro-9-(*tert*-pentyl)-9*H*-purine (**5**)

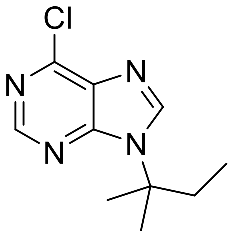

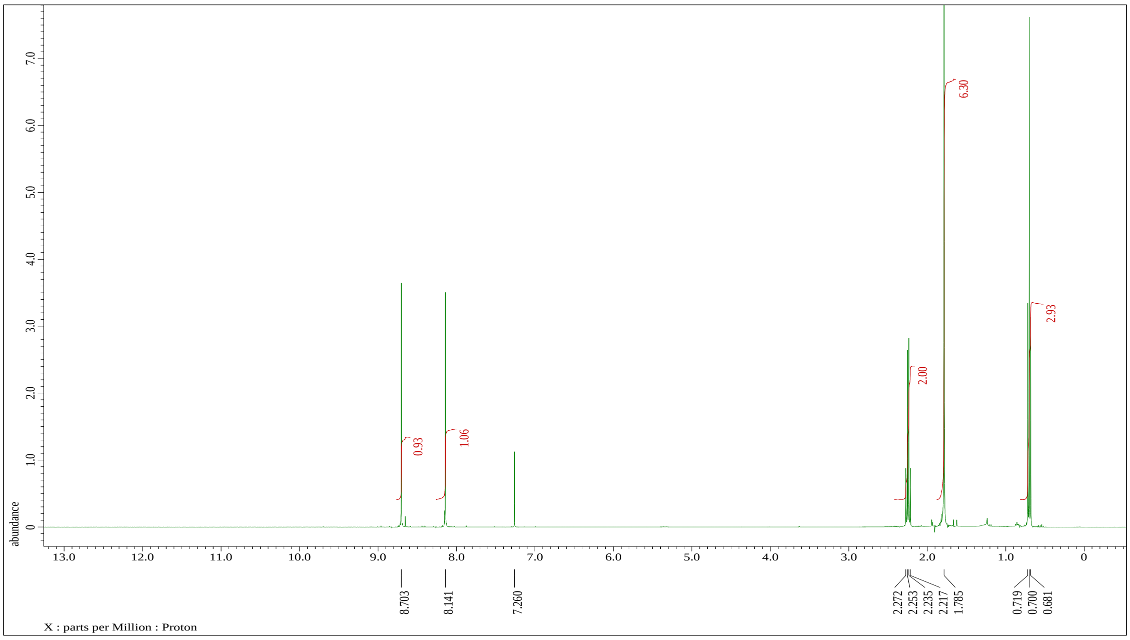

^{13}C{^{1}H} NMR (101 MHz, $CDCl_3$)

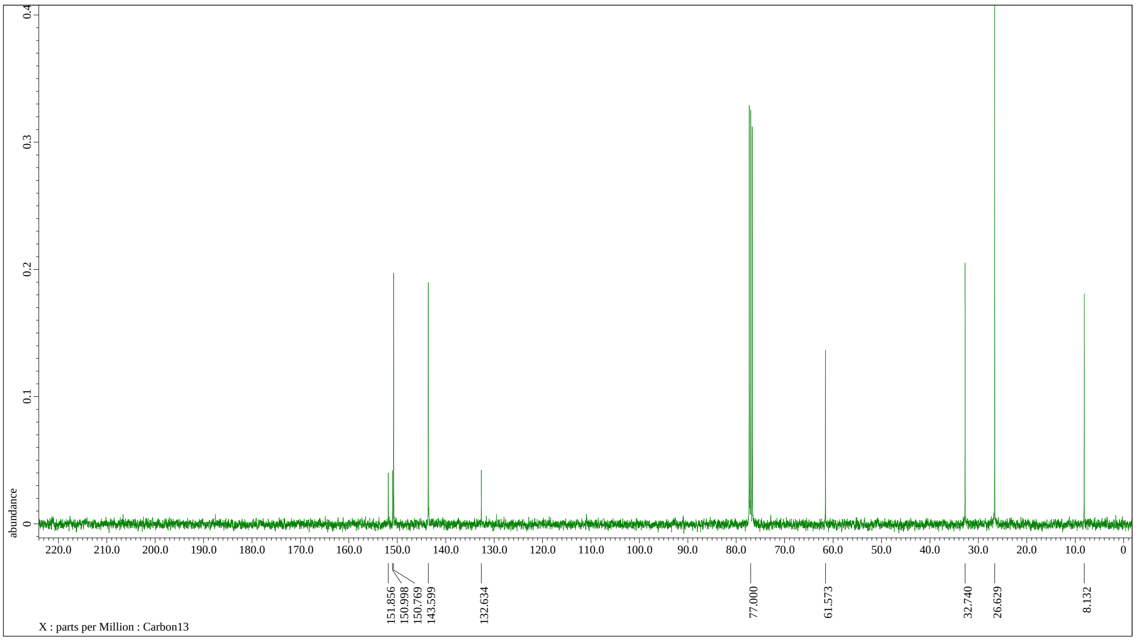

^{1}H NMR (400 MHz, $CDCl_3$) 7-(*tert*-butyl)-6-chloro-2-(methylthio)-7*H*-purine (**7**)

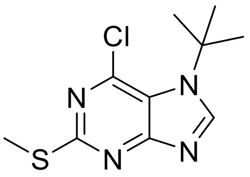

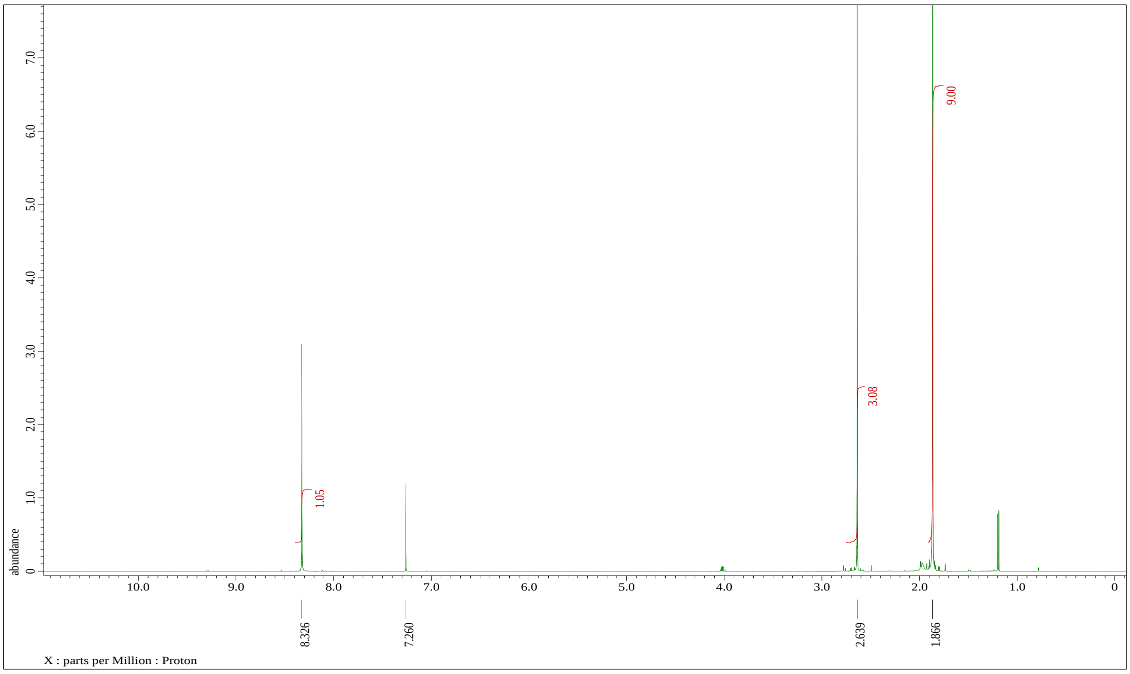

^{13}C{^{1}H} NMR (101 MHz, $CDCl_3$)

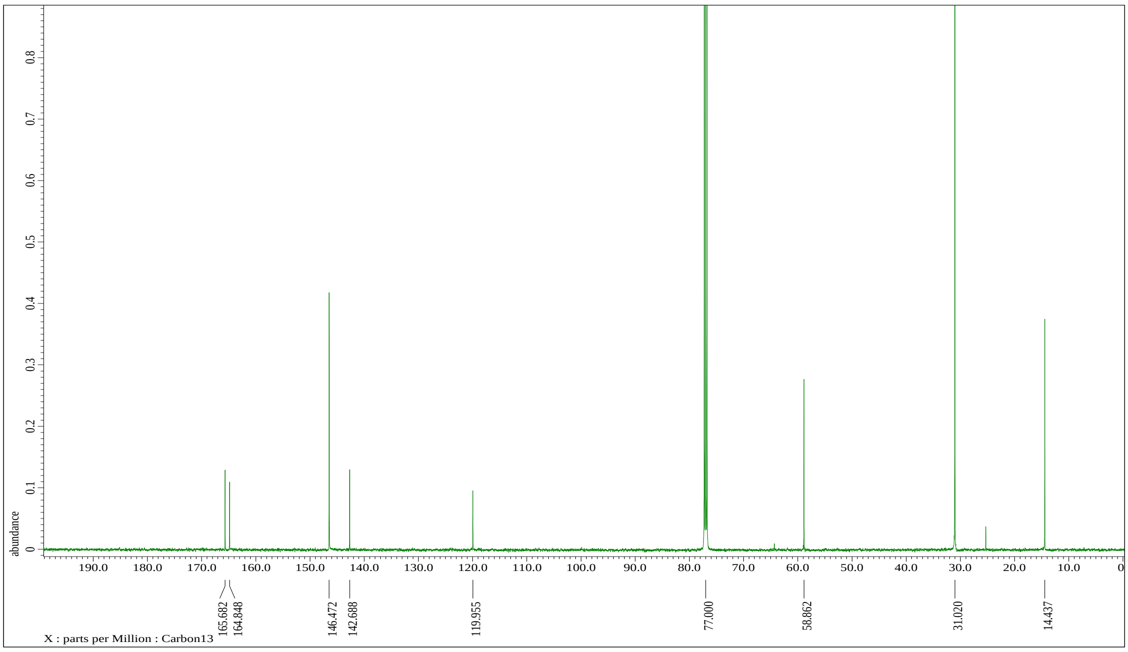

^{1}H NMR (400 MHz, $CDCl_3$) **9**-(*tert*-butyl)-6-chloro-2-(methylthio)-9*H*-purine (**9**)

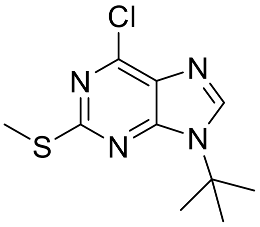

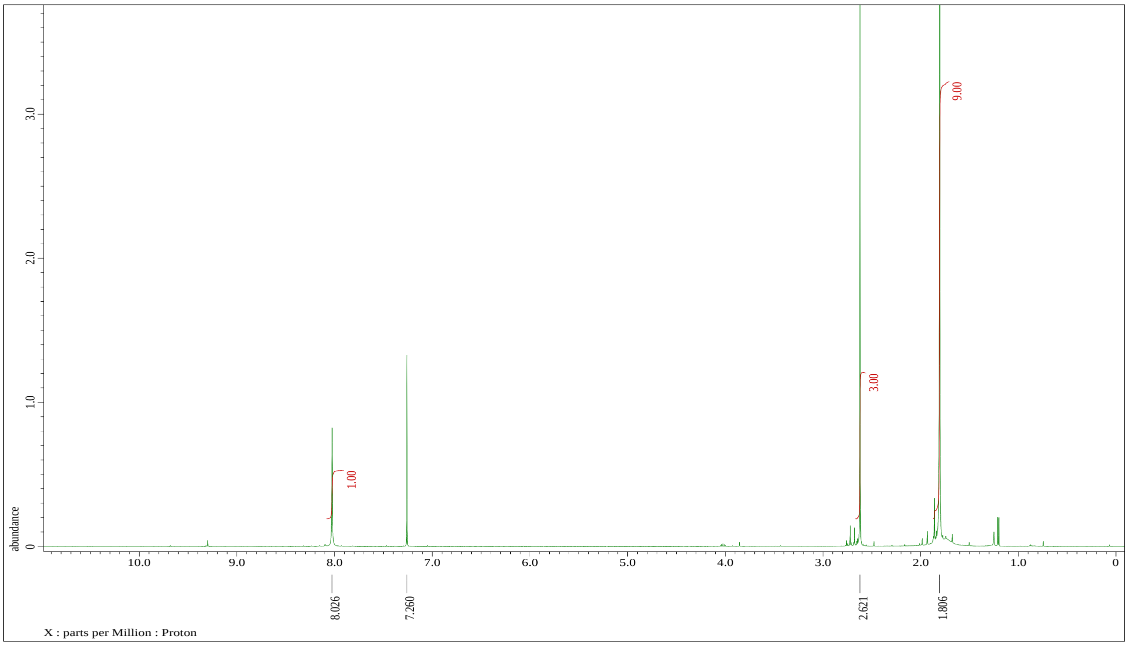

^{13}C{^{1}H} NMR (101 MHz, $CDCl_3$)

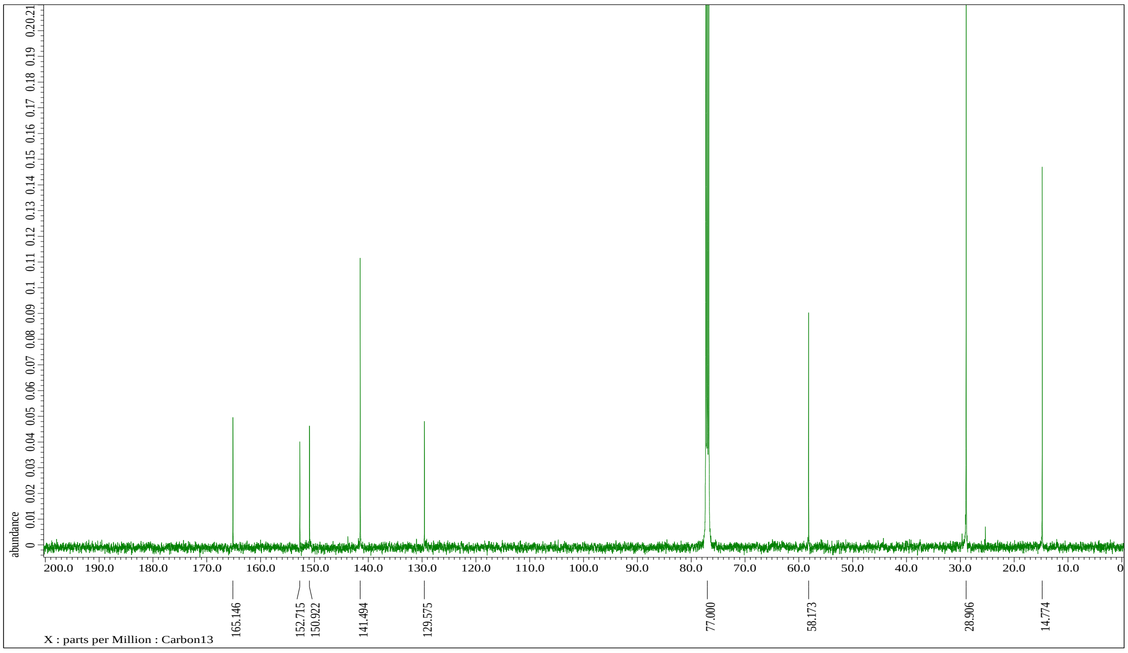

^{1}H NMR (400 MHz, $CDCl_3$) 7-(*tert*-butyl)-6-methoxy-7*H*-purine (**11**)

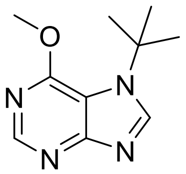

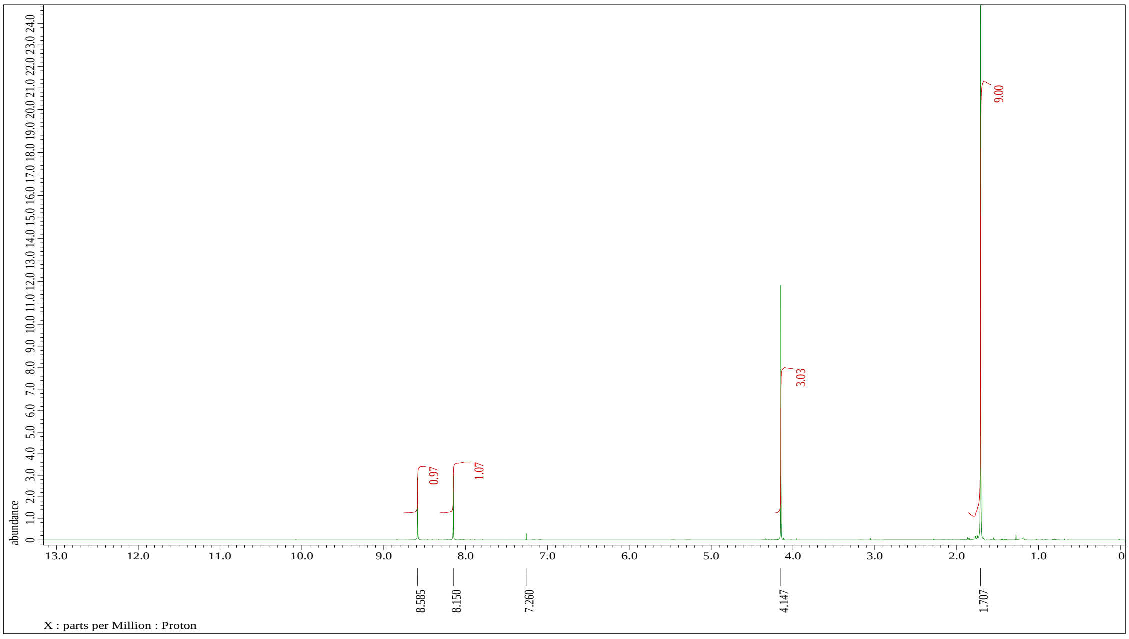

^{13}C NMR (101 MHz, $CDCl_3$)

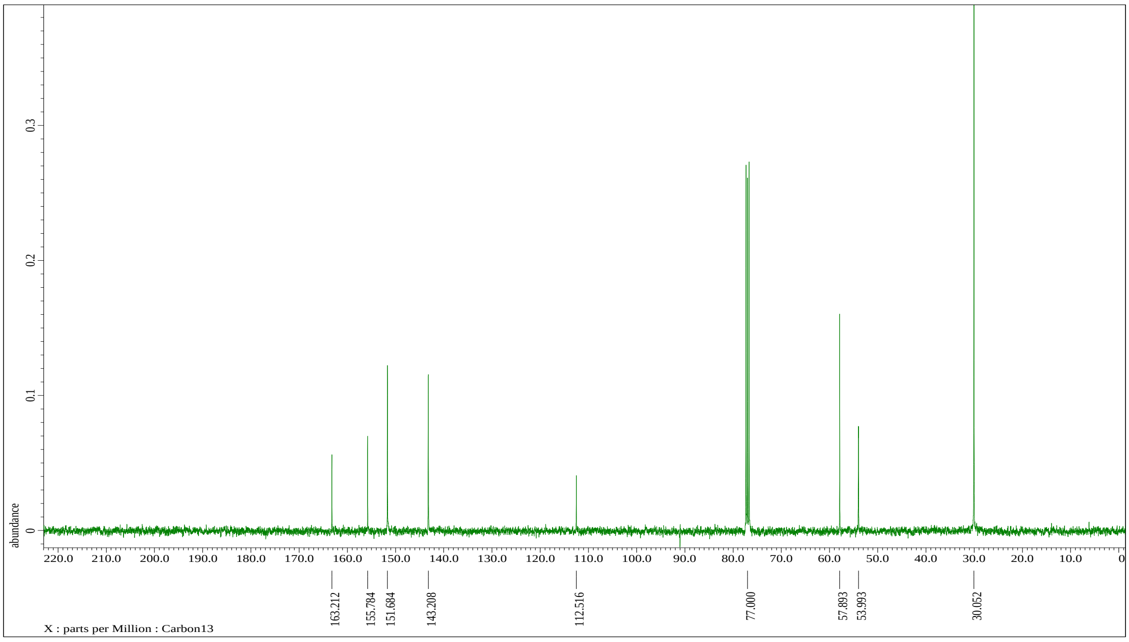

^{1}H NMR (400 MHz, $CDCl_3$) 7-(*tert*-butyl)-6-(methylthio)-7*H*-purine (**12**)

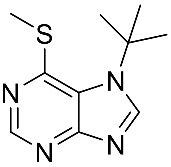

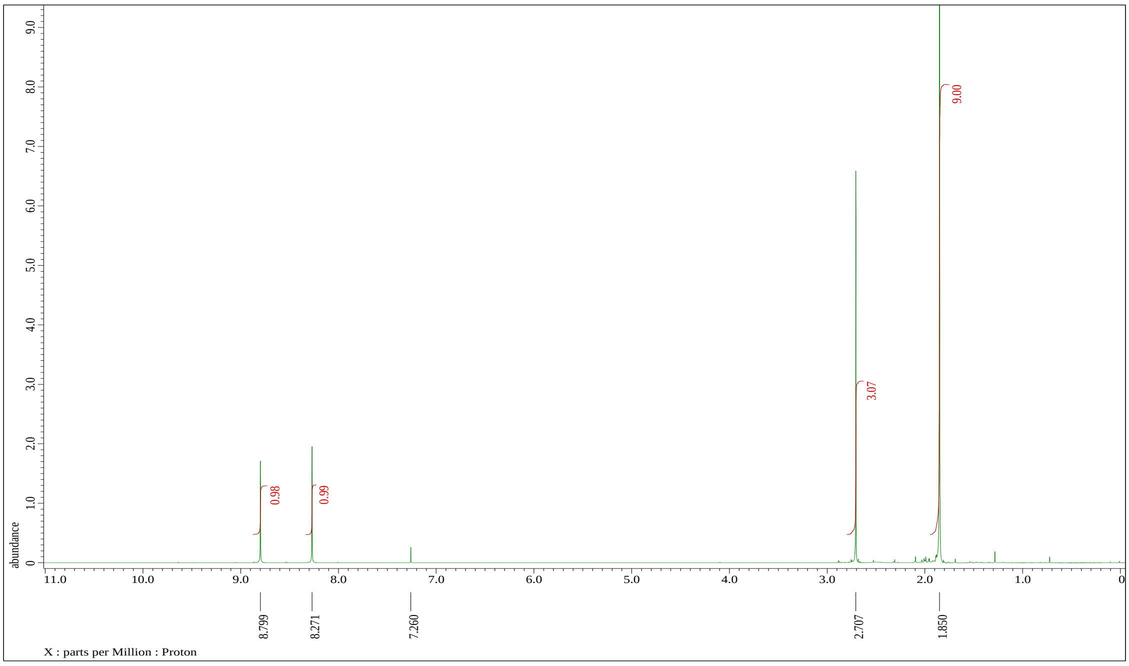

^{13}C{^{1}H} NMR (101 MHz, $CDCl_3$)

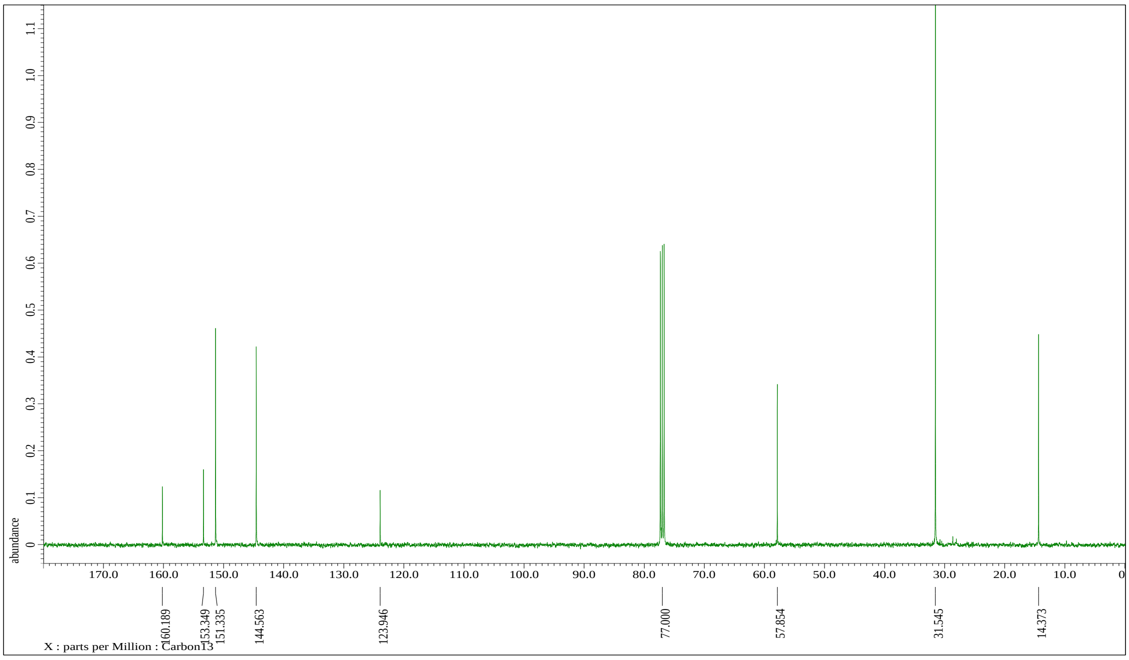

^{1}H NMR (400 MHz, $CDCl_3$) 7-(*tert*-butyl)-7*H*-purin-6-ol (**13**)

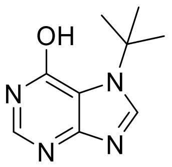

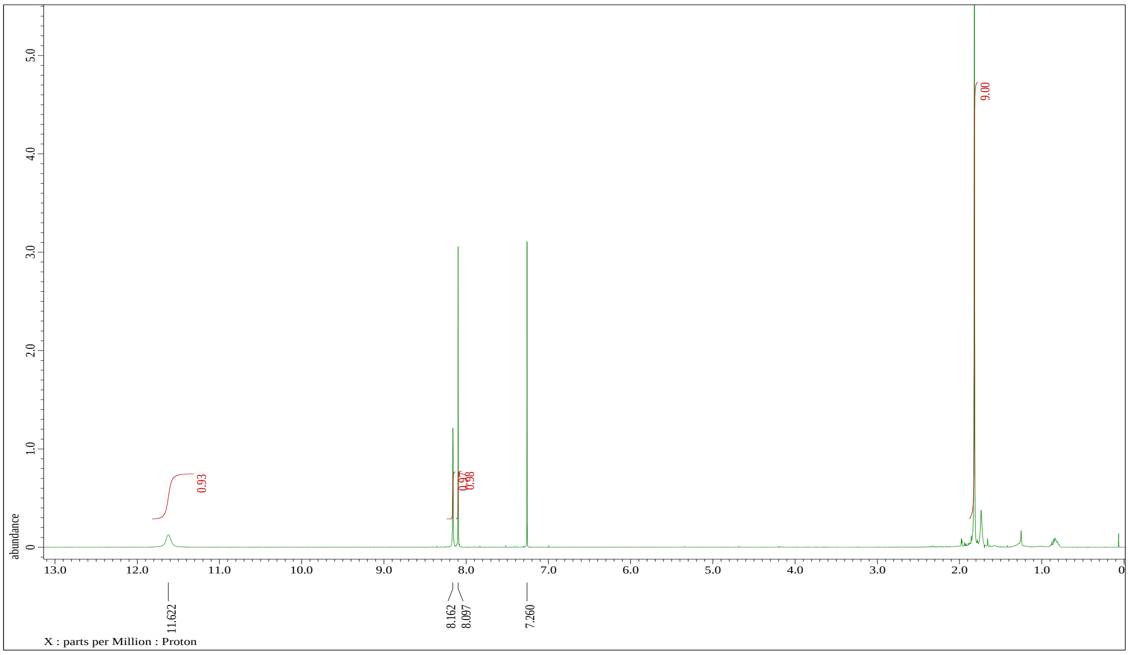

^{13}C{^{1}H} NMR (101 MHz, $CDCl_3$)

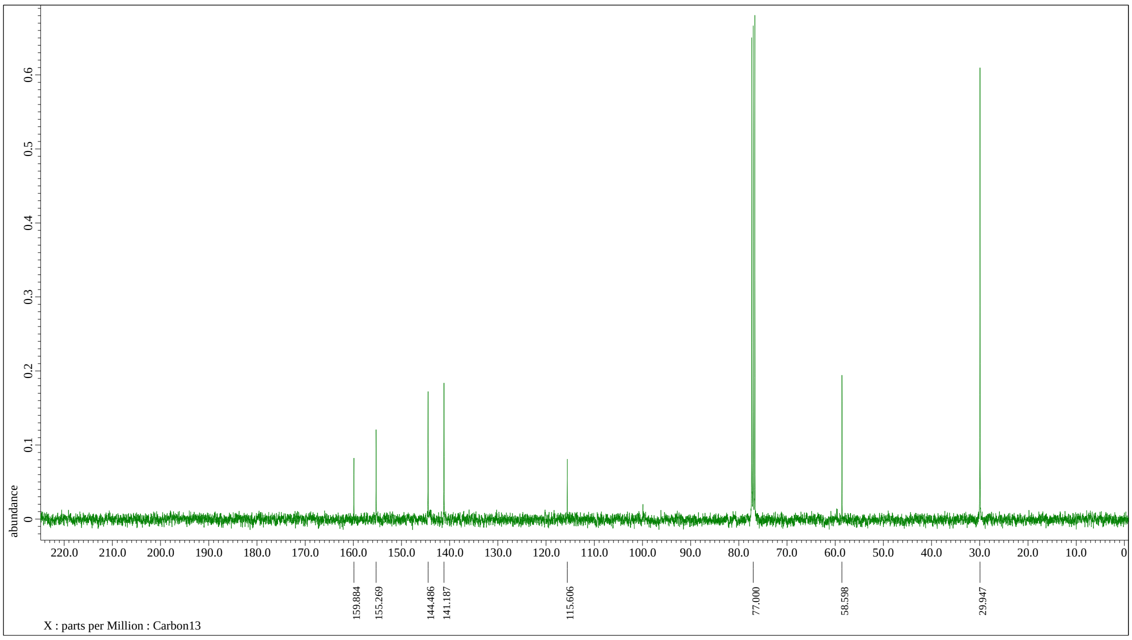

^{1}H NMR (400 MHz, $CDCl_3$) 7-(*tert*-butyl)-6-ethoxy-7*H*-purine (**14**)

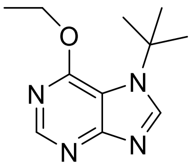

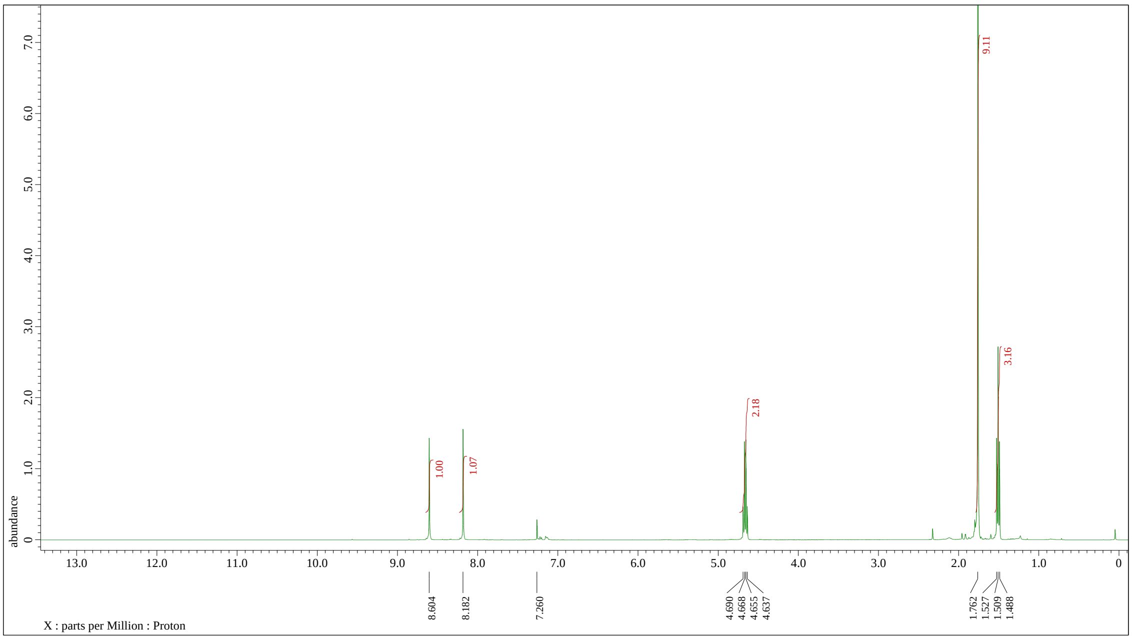

^{13}C{^{1}H} NMR (101 MHz, $CDCl_3$)

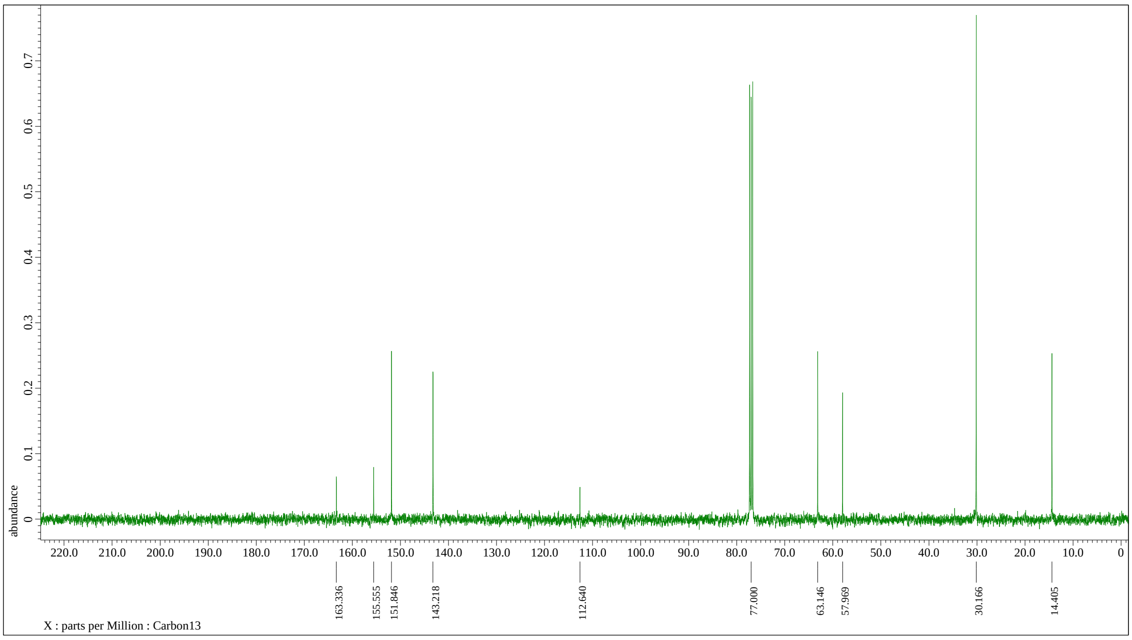

^{1}H NMR (400 MHz, DMSO-d_6) 7-(*tert*-butyl)-1,7-dihydro-6*H*-purin-6-thione (**15**)

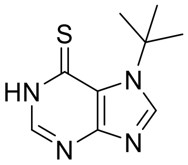

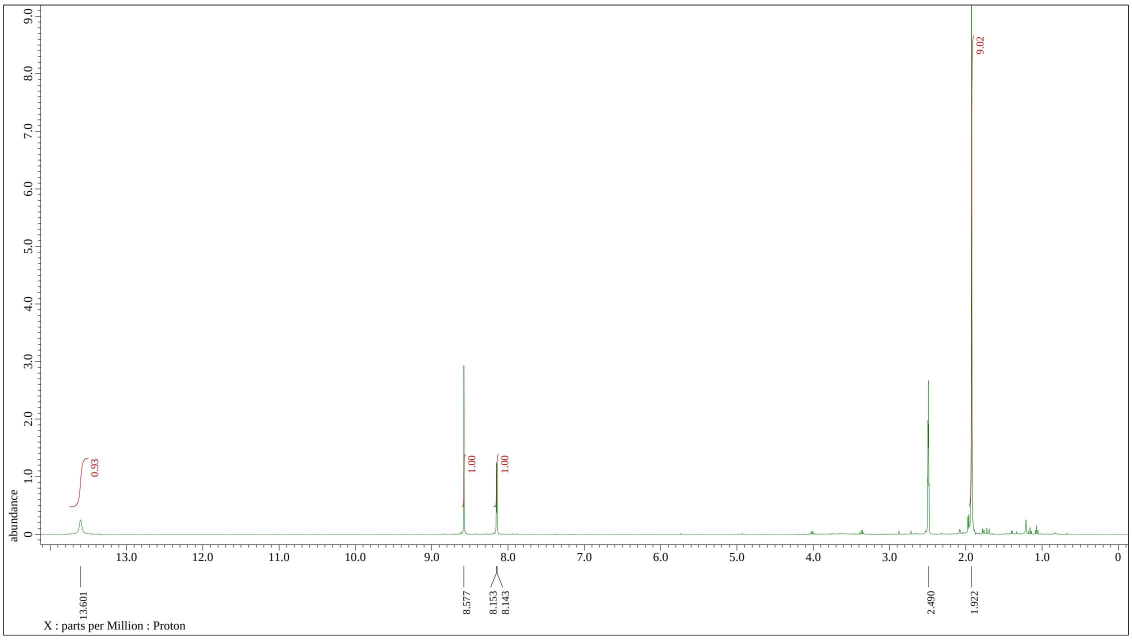

^{13}C{^{1}H} NMR (101 MHz, DMSO-d_6)

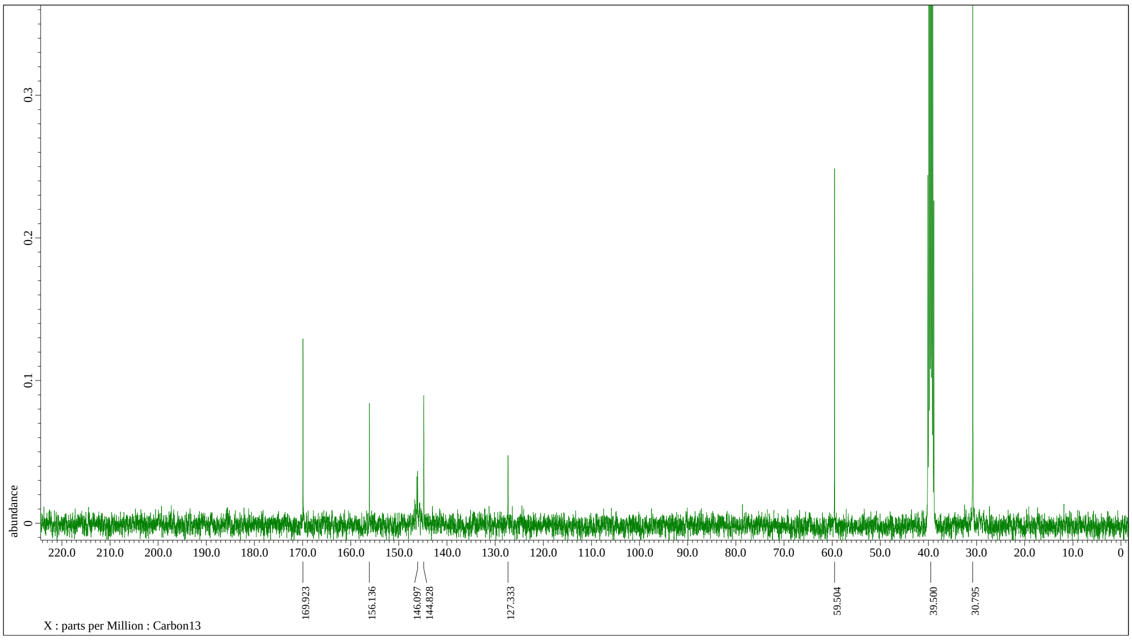

^{1}H NMR (400 MHz, $CDCl_3$) 6-azido-7-(*tert*-butyl)-7*H*-purine (**16**)

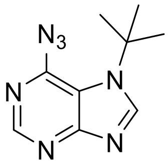

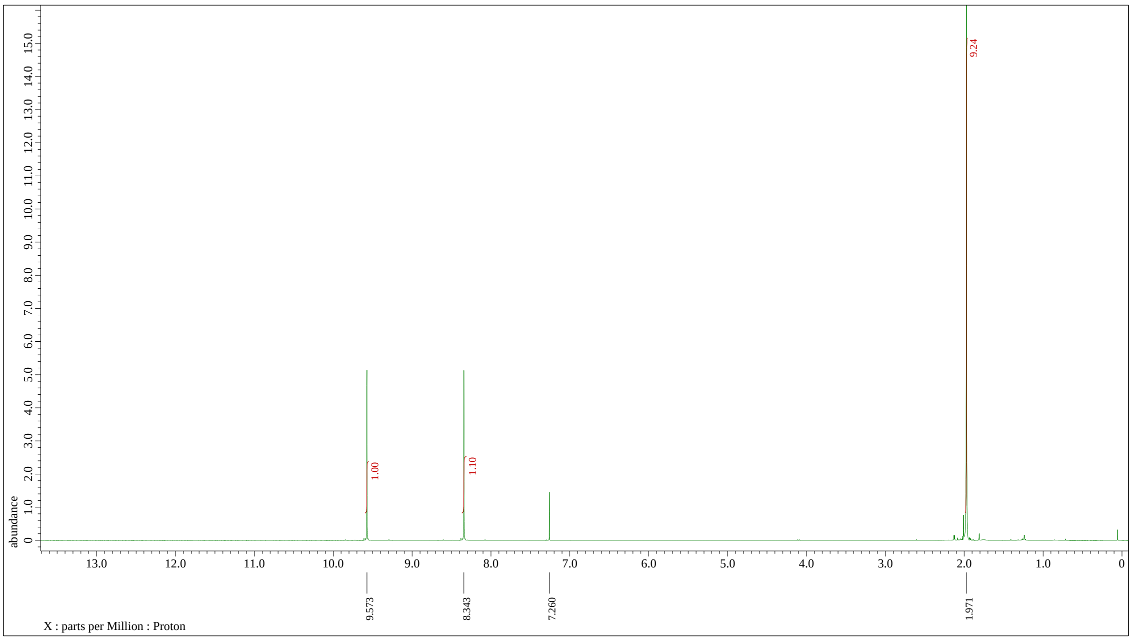

$^{13}C\{^{1}H\}$ NMR (101 MHz, $CDCl_3$)

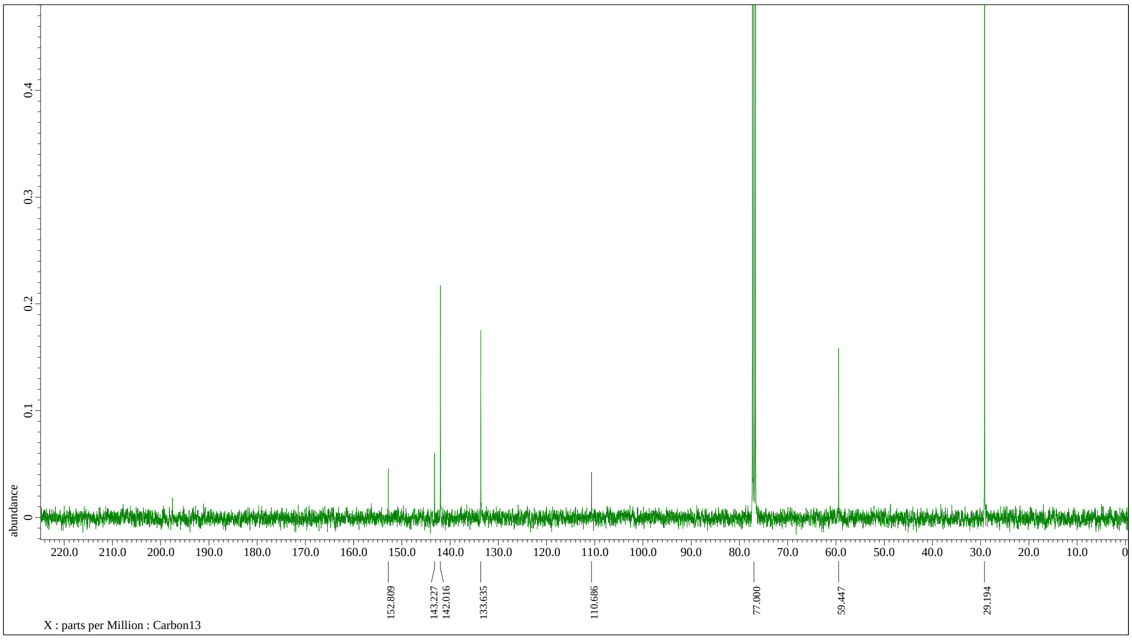

^{1}H NMR (400 MHz, DMSO-d_6) 7-(*tert*-butyl)-7*H*-purin-6-amine (**17**)

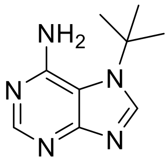

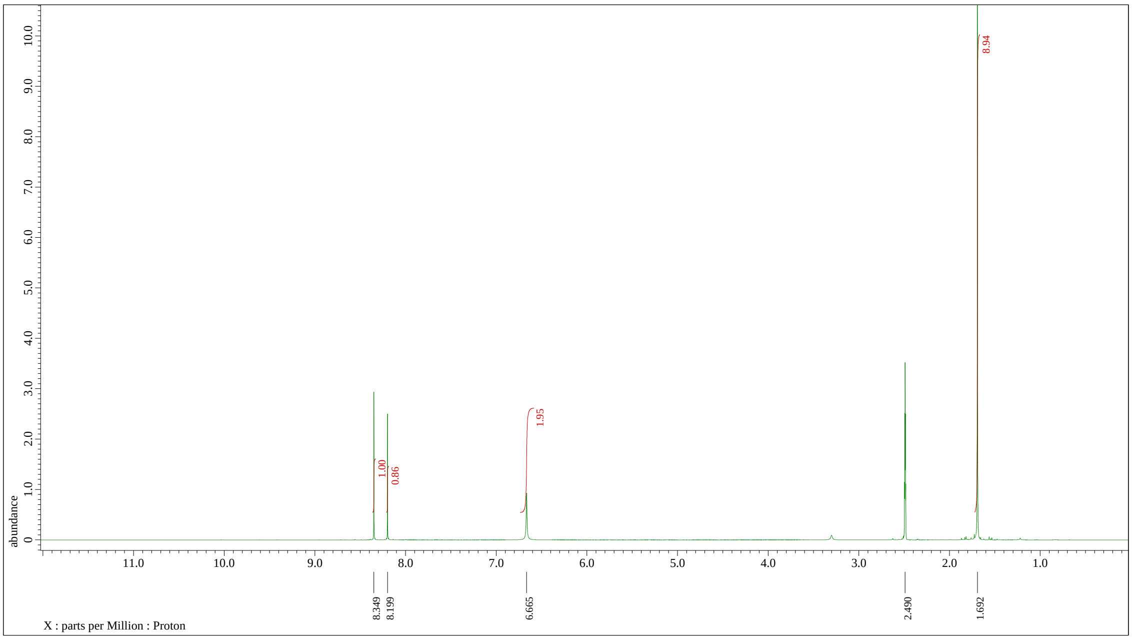

^{13}C{^{1}H} NMR (101 MHz, DMSO-d_6)

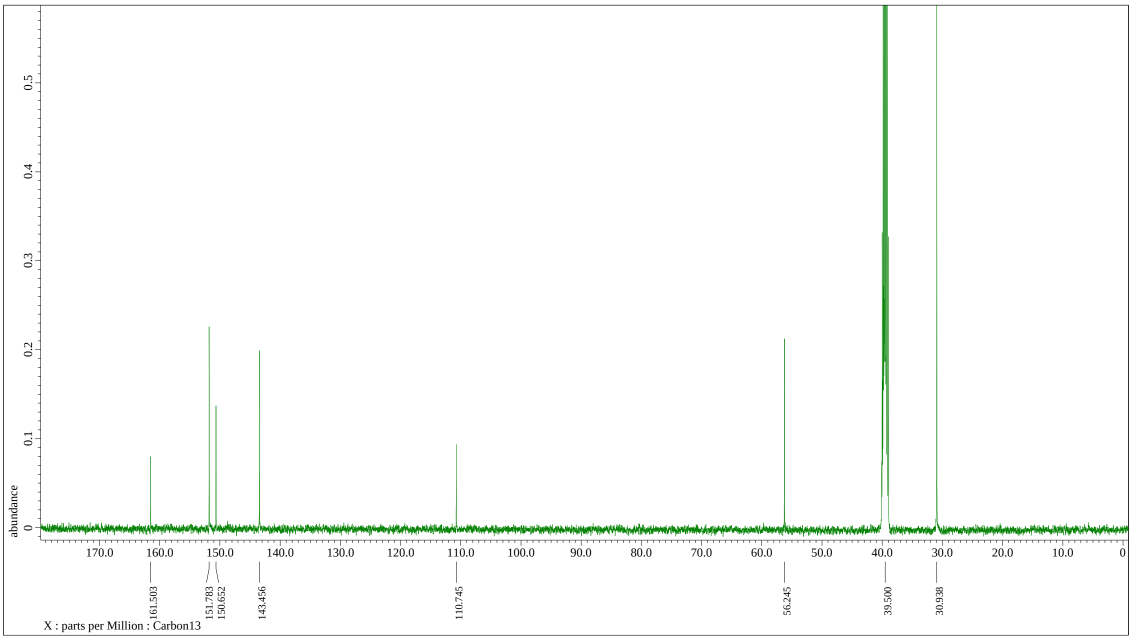

^{1}H NMR (400 MHz, $CDCl_3$) *N*-benzyl-7-(*tert*-butyl)-7*H*-purin-6-amine (**18**)

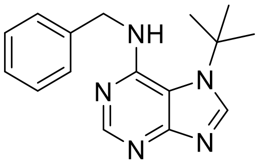

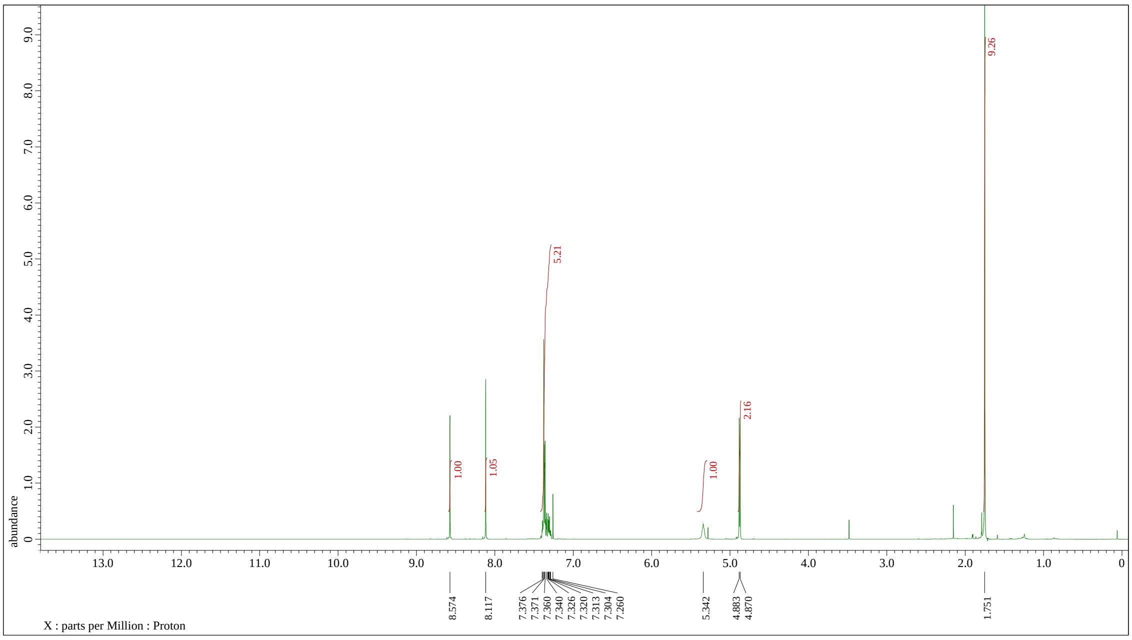

^{13}C{^{1}H} NMR (101 MHz, $CDCl_3$)

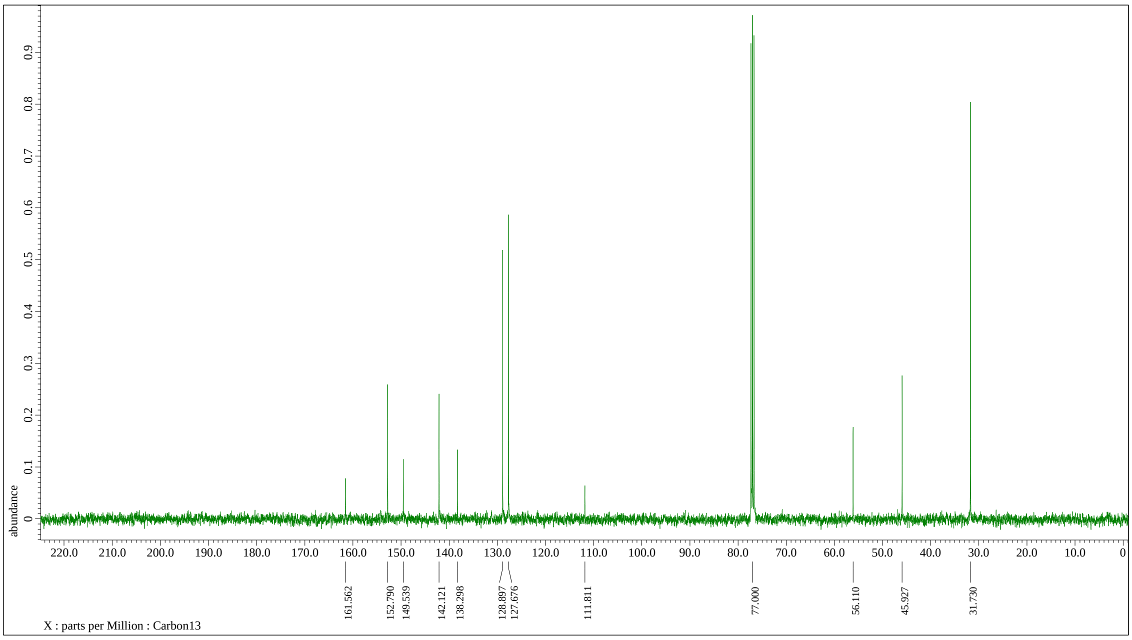

^{1}H NMR (400 MHz, $CDCl_3$) 7-(*tert*-butyl)-*N*-cyclohexyl-7*H*-purin-6-amine (**19**)

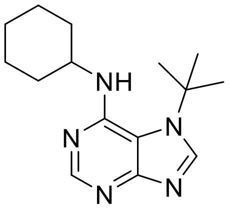

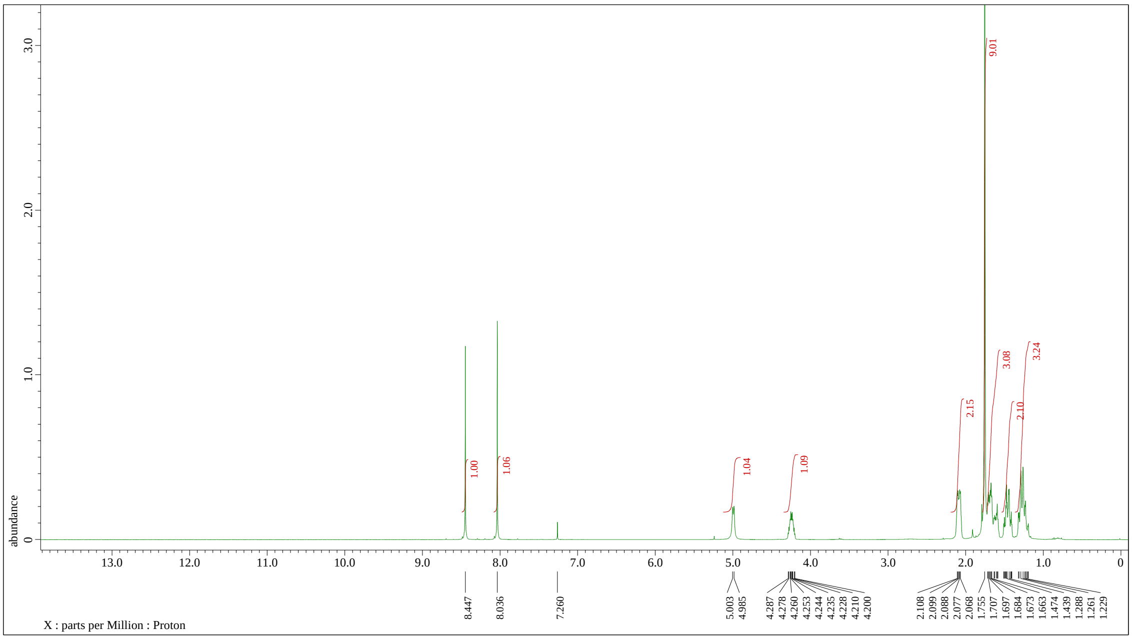

^{13}C{^{1}H} NMR (101 MHz, $CDCl_3$)

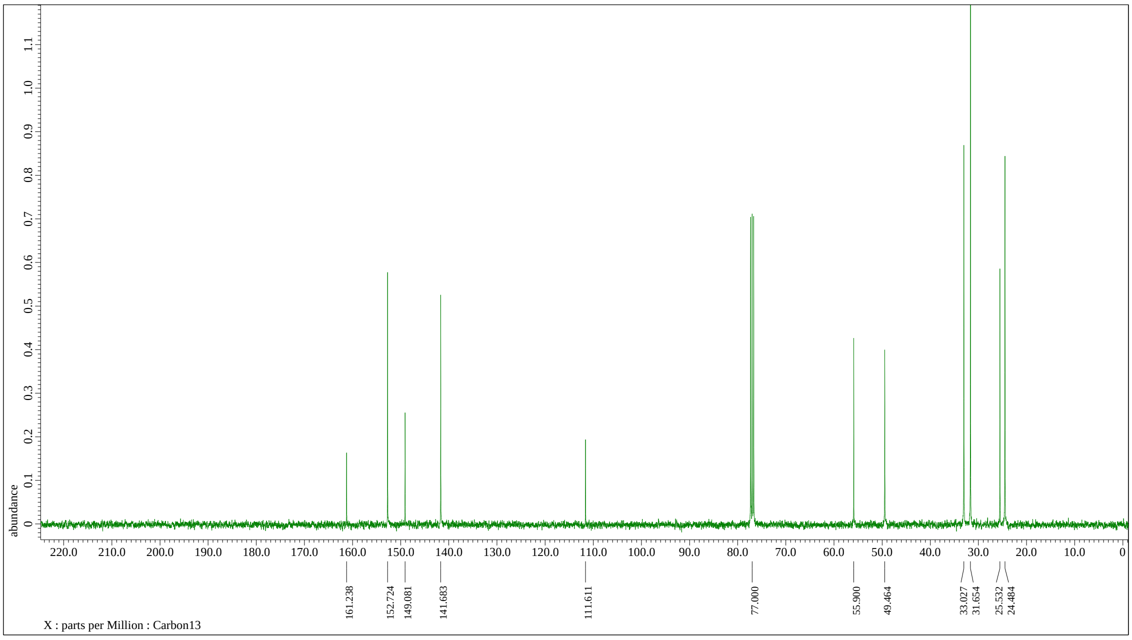

^{1}H NMR (400 MHz, $CDCl_3$) 3-(((7-(*tert*-butyl)-7*H*-purin-6-yl)amino)methyl)phenol (**20**)

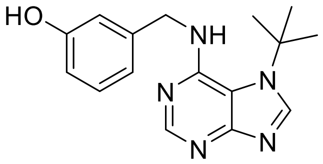

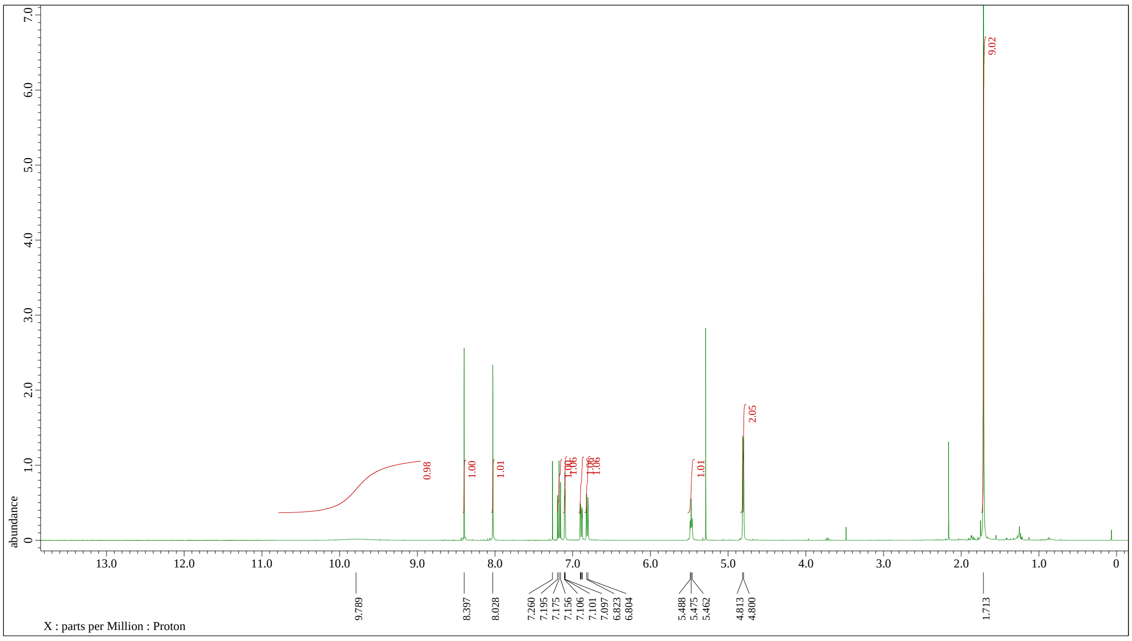

^{13}C{^{1}H} NMR (101 MHz, $CDCl_3$)

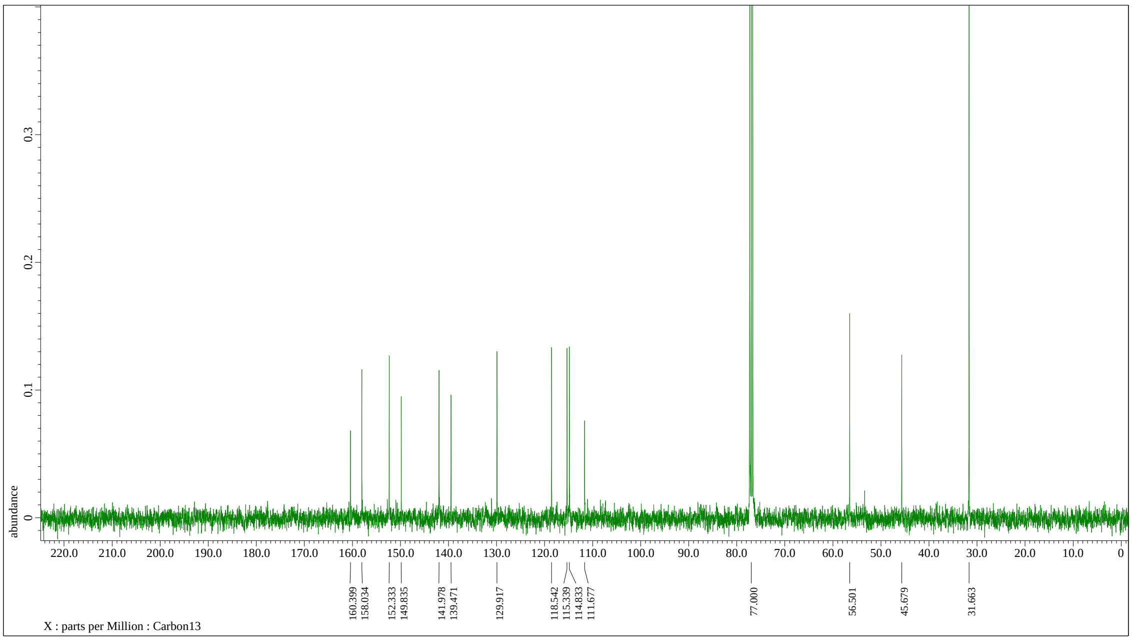

^{1}H NMR (400 MHz, $CDCl_3$) 7-(*tert*-butyl)-*N*-(furan-2-ylmethyl)-7*H*-purin-6-amine (**21**)

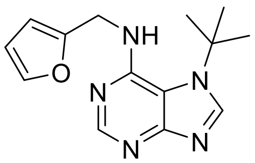

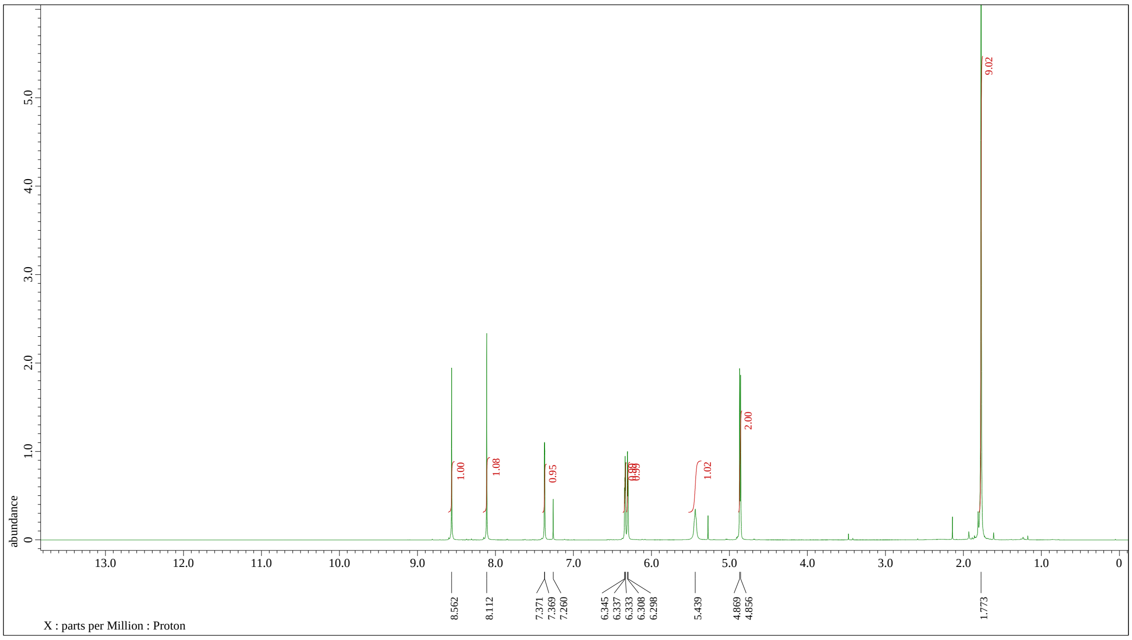

$^{13}C\{^1H\}$ NMR (101 MHz, $CDCl_3$)

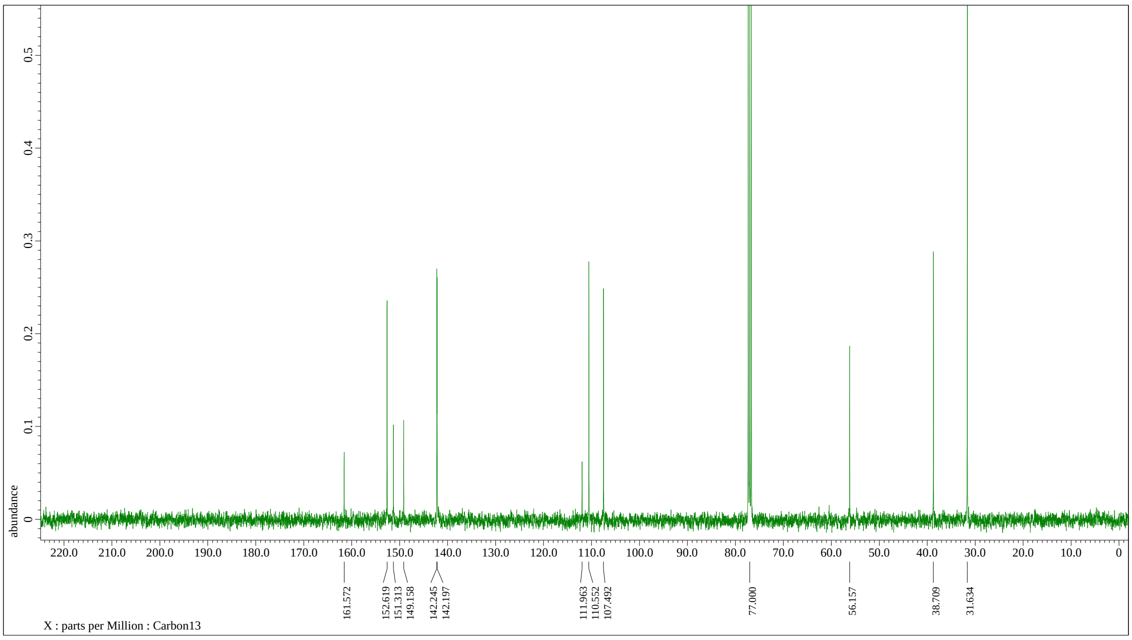

^{1}H NMR (400 MHz, $CDCl_3$) 7-(*tert*-butyl)-6-(piperidin-1-yl)-7*H*-purine (**22**)

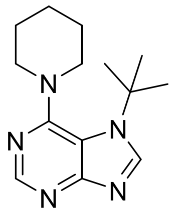

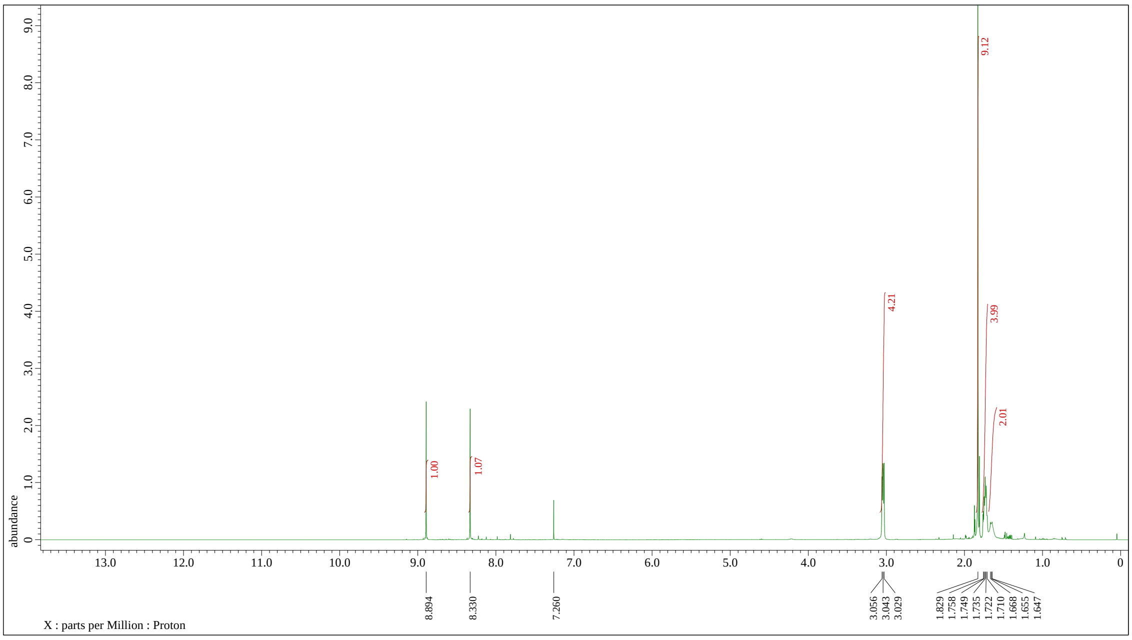

$^{13}C\{^{1}H\}$ NMR (101 MHz, $CDCl_3$)

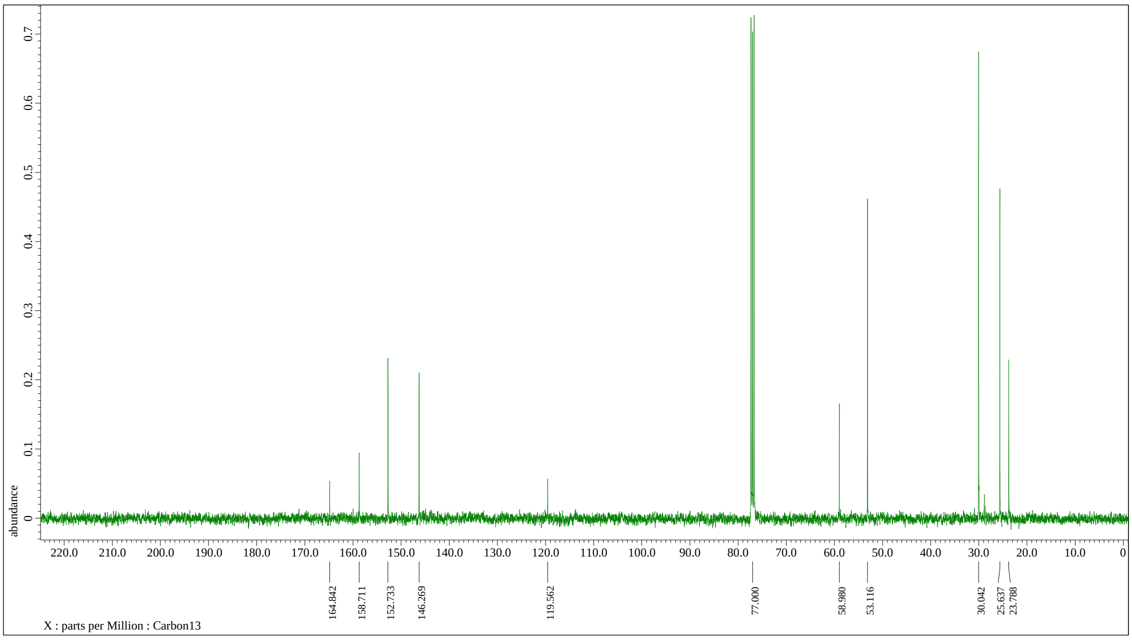

^{1}H NMR (400 MHz, $CDCl_3$) 7-(*tert*-butyl)-6-(4-methoxyphenyl)-7*H*-purine (**23**)

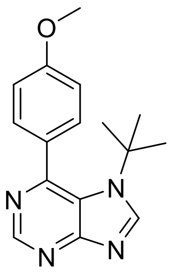

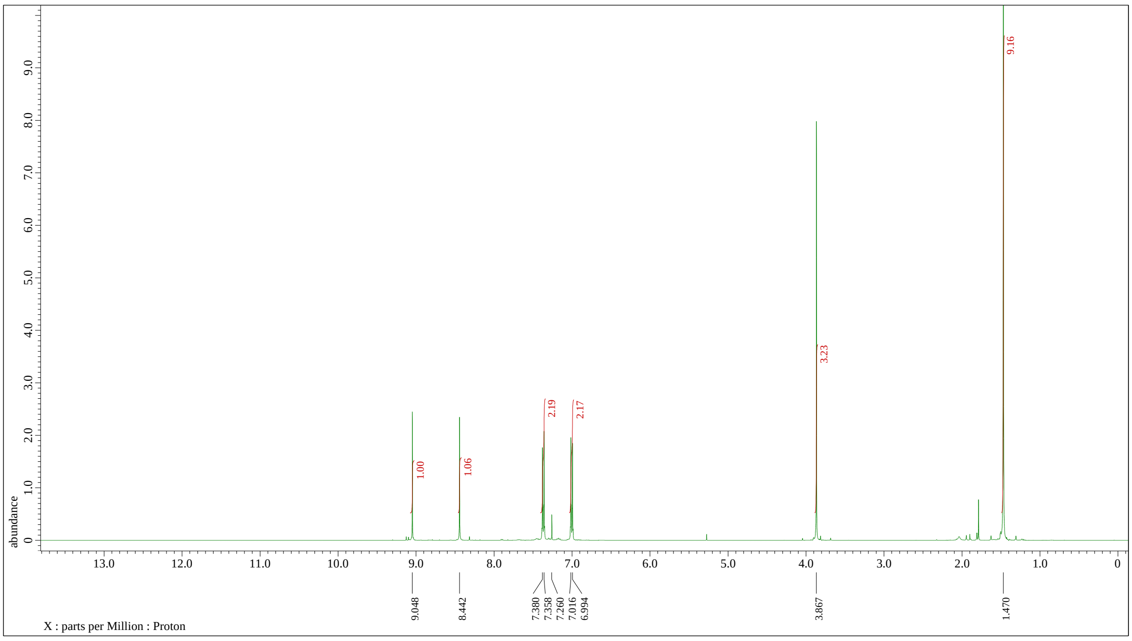

^{13}C NMR (101 MHz, $CDCl_3$)

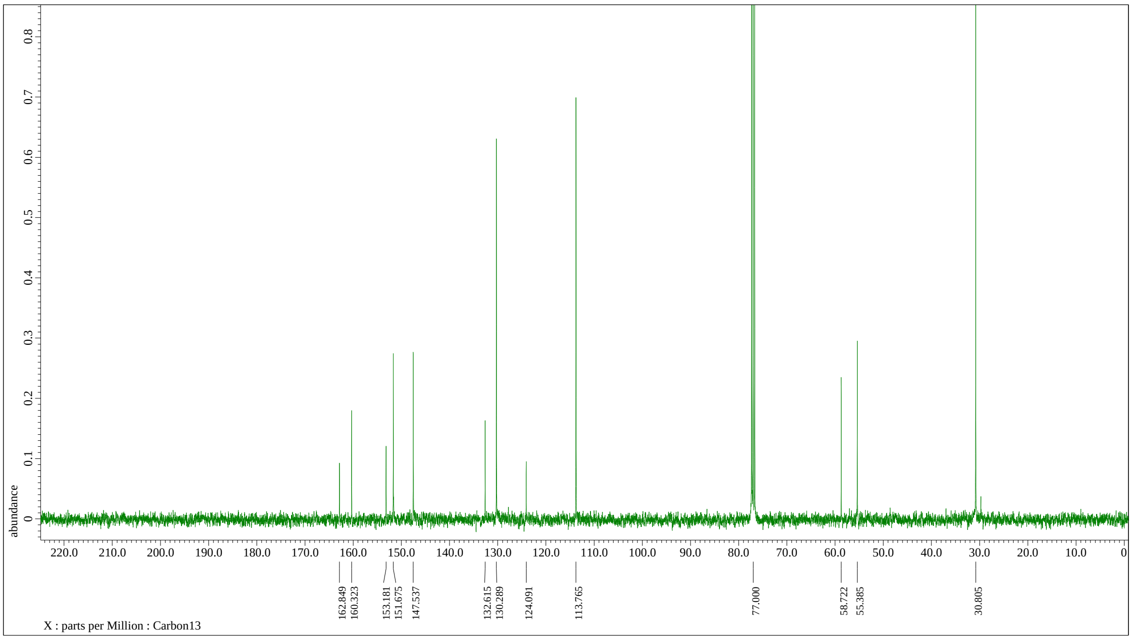

^{1}H NMR (400 MHz, $CDCl_3$) 7-(*tert*-butyl)-6-(thiophen-3-yl)-7*H*-purin (**24**)

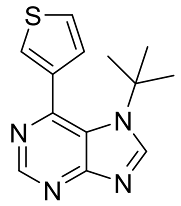

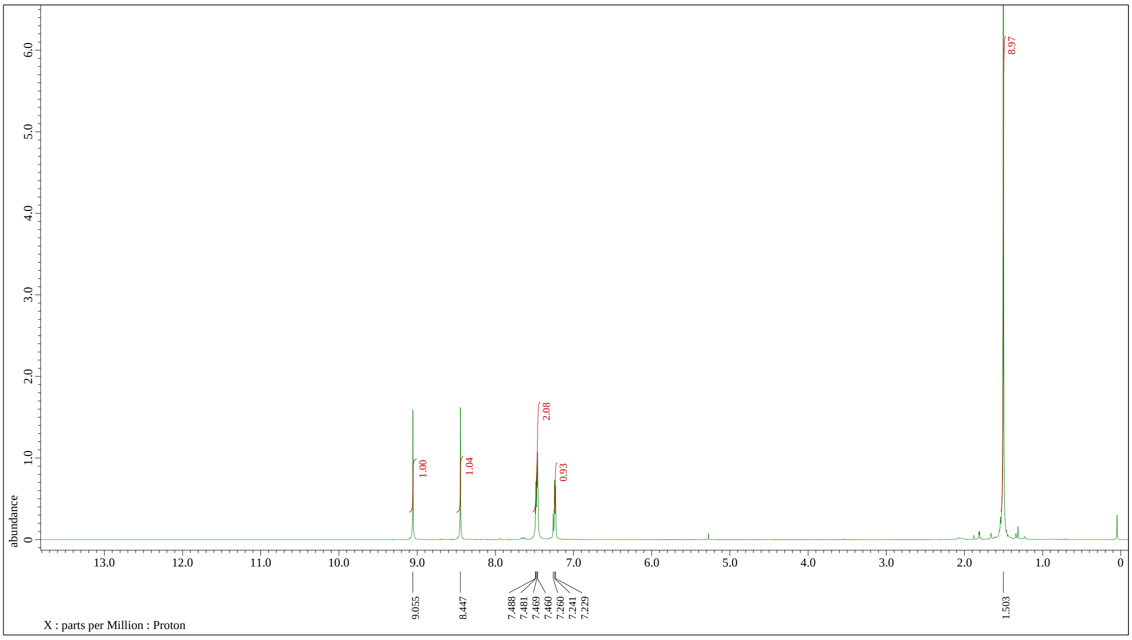

^{13}C{^{1}H} NMR (101 MHz, $CDCl_3$)

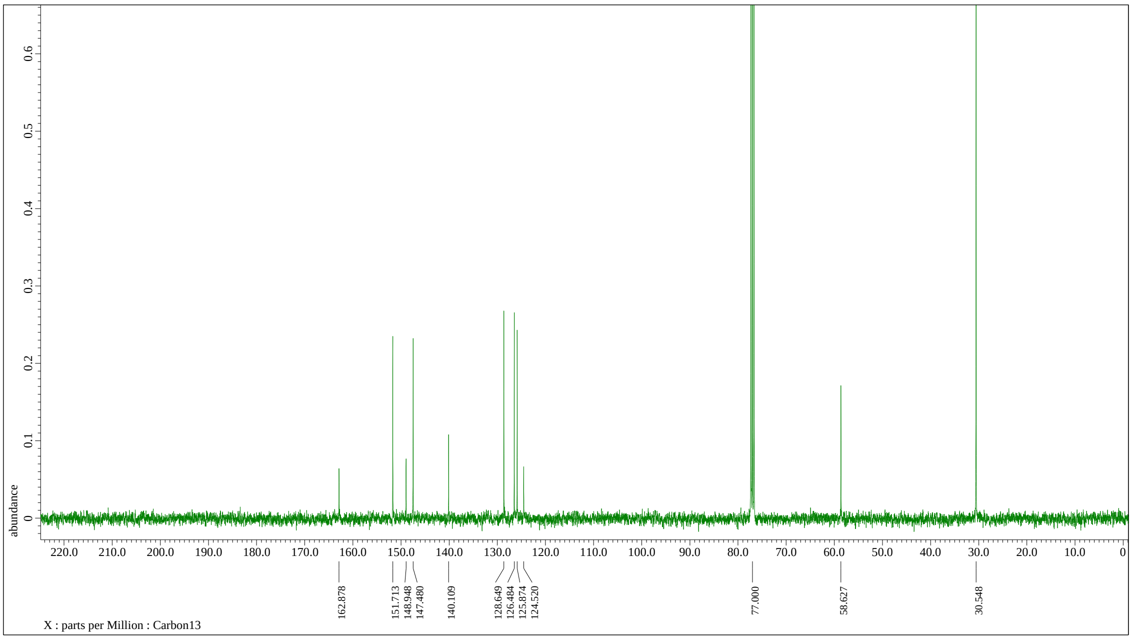

NOESY NMR spectrum of **11**

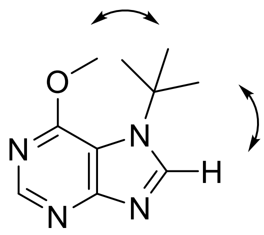

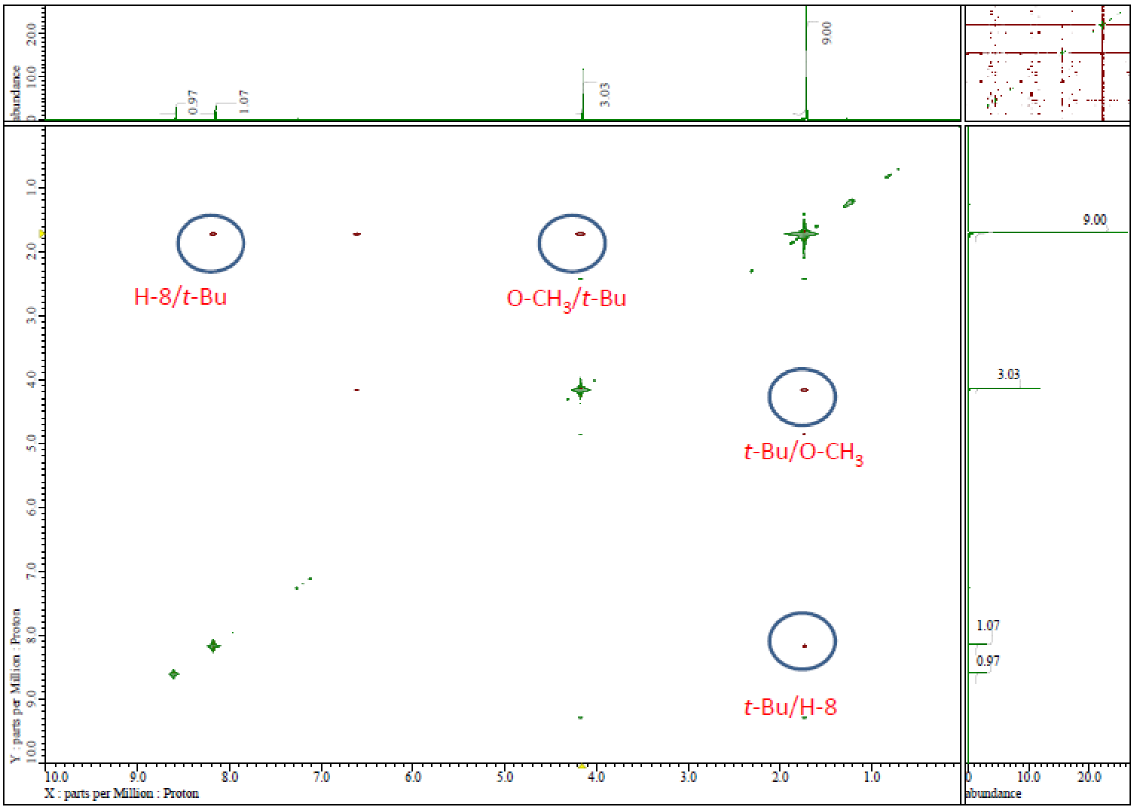

NOESY NMR spectrum of **12**

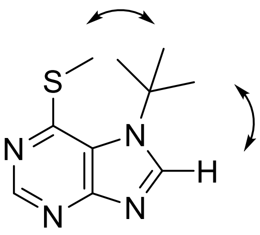

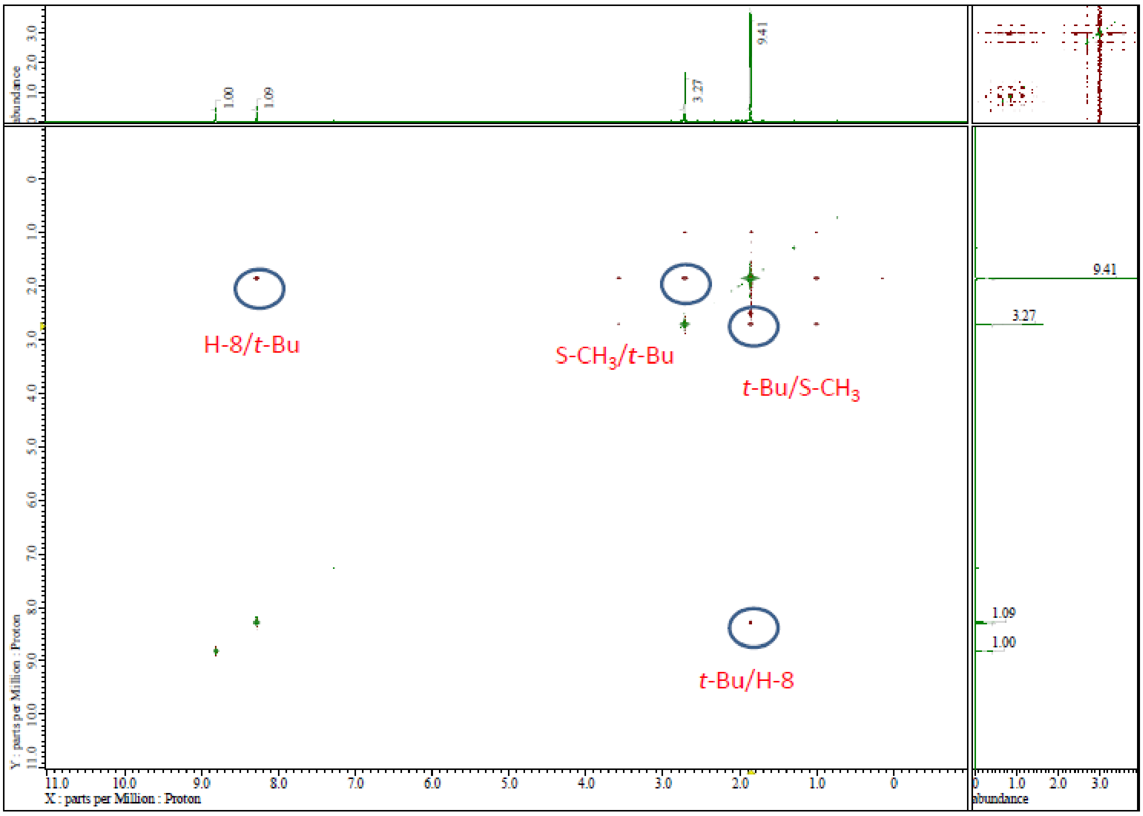

HPLC analysis of the *tert*-butylation reaction of 6-chloropurine in acetonitrile, Table 1, entry 18 and 25

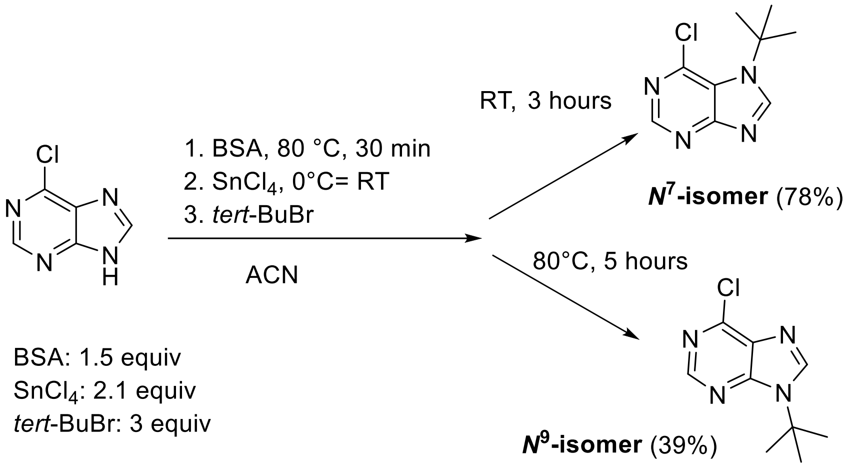

Analysis of the reaction mixture at **RT for 3 hours (Table 1, entry 18)**

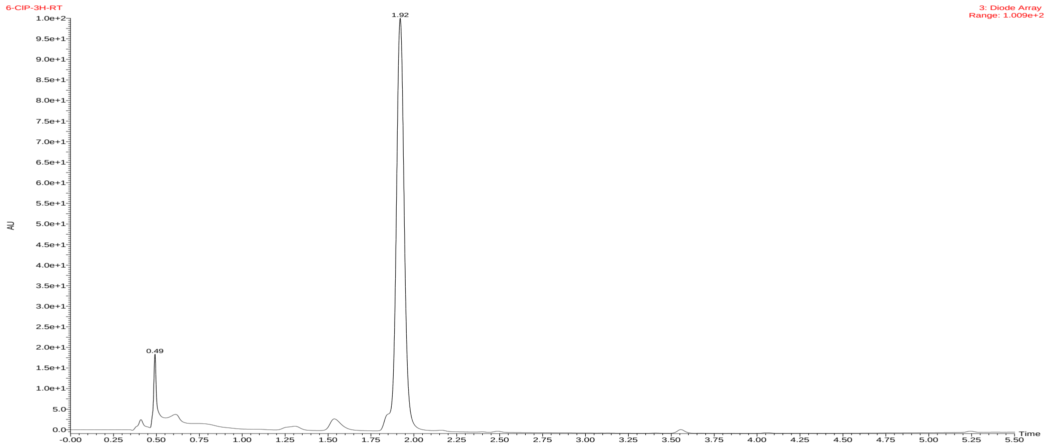

Analysis of the reaction mixture **at 80 °C for 5 hours (Table 1, entry 25)**

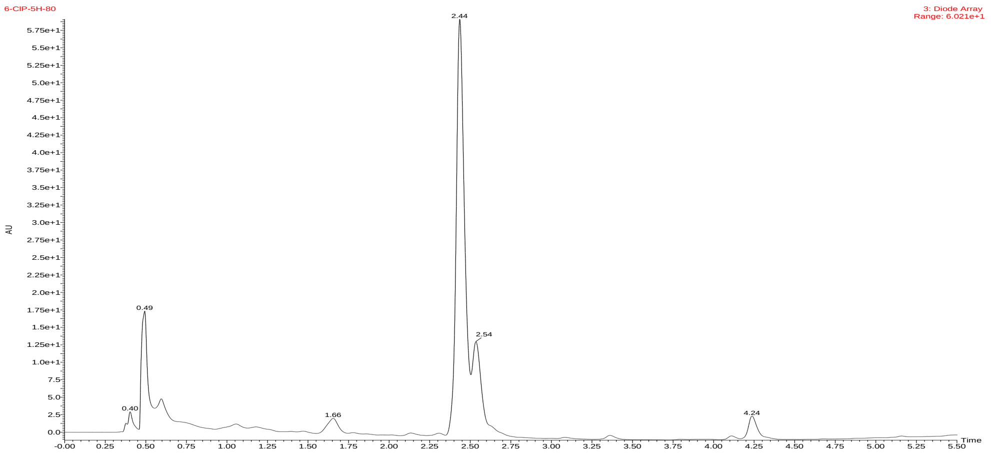

Supplement: Supplementary file 1 — ao4c00068_si_001.pdf [file ao4c00068_si_001.pdf]
